# Errors, discrepancies and underlying bias in radiology with case examples: a pictorial review

**DOI:** 10.1186/s13244-021-00986-8

**Published:** 2021-04-20

**Authors:** Omer Onder, Yasin Yarasir, Aynur Azizova, Gamze Durhan, Mehmet Ruhi Onur, Orhan Macit Ariyurek

**Affiliations:** Department of Radiology, Hacettepe University School of Medicine, Ankara, 06100 Turkey

**Keywords:** Medical errors, Bias, Diagnostic errors, Diagnostic ımaging, Radiology

## Abstract

Interpretation differences between radiologists and diagnostic errors are significant issues in daily radiology practice. An awareness of errors and their underlying causes can potentially increase the diagnostic performance and reduce individual harm. The aim of this paper is to review both the classification of errors and the underlying biases. Case-based examples are presented and discussed for each type of error and bias to provide greater clarity and understanding.

## Key points

- Errors, discrepancies and confounding biases are inseparable parts of radiology practice with various clinical consequences.
- Radiological errors can occur before, during or after the reporting periods.
- Effective communication between radiologists, radiology technicians, patients and clinicians is the key to proper patient management
- Being familiar with the types of errors and underlying biases is essential for radiologists to cope with them.

## Background

Radiological imaging is an essential part of patient management. Despite significant technological developments, a radiological investigation is rarely definitive on its own, leading to discrepancies between radiological impressions and the ultimate outcome. Moreover, radiology reports, like all human endeavours, may contain errors or misunderstandings. The term "error" is described as no uncertainty about the correct finding, with no possibility for dispute or disagreement, while the word "discrepancy" stands for justifiable differences of opinion between colleagues [1–3].

Errors and discrepancies may cause direct or indirect, permanent, or temporary harmful effects because of a false, missed, or delayed diagnosis, or may not result in any harm if clinically insignificant, or feedback is received from clinicians or other radiologists [4, 5].

In the literature, the classification of radiological errors and underlying biases has been discussed by different authors. Radiological errors can be classified according to the reporting process as pre-reporting, reporting or post-reporting errors. Pre-reporting errors consist of technical issues and procedure-related problems, whereas post-reporting errors are mainly caused by poor communication between radiologists and clinicians. Reporting errors are directly related to radiologists and can be categorized into two parts. "Perceptual errors" are more common and related to the fact that the present finding is not noticed, while "interpretative errors" are influenced by cognitive biases that can contribute to false reasoning. The classification of radiological error types according to the reporting process is shown in Table 1 [3, 4, 6].

**Table 1:** Classification of radiological error types according to the reporting process

Pre-reportıng errors		Reportıng errors (related to the radıologıst)		Post-reportıng errors
Technique-related (type 6) errors	Procedure-related (type 11) errors	Perceptual errors	Interpretative errors	
Inappropriate choice of study/modality	Complications	Underreading (type 4) error	Overreading (type 1) error	Poor communication **(type 5)** error
Incomplete study	Contrast agent extravasation	Location (type 9) error	Faulty reasoning (type 2) error	Absence of needed impression and recommendation (due to the radiologist)
Missing sequences	Allergic reactions	Satisfaction of search (type 10) error	Lack of knowledge (type 3) error	Reporter error at the time of report writing
Study without contrast	Nephrotoxicity	Prior examination (type 7), History (type 8) and Satisfaction of report (type 12) errors may be included in both perceptual and interpretative errors, depend on the situation		Clinicians may not understand or may misunderstand the report
Suboptimal study	Interventional radiology procedures			
Artifacts				
Poor technique				

According to the comprehensive classification system of Kim-Mansfield, there are 12 subgroups defined for radiological error types [7]. In this paper, the classifications are reviewed, and cases are presented related to those 12 types of radiological errors and underlying bias.

### Definitions of “error” and “discrepancy”

**Fig. 1:**
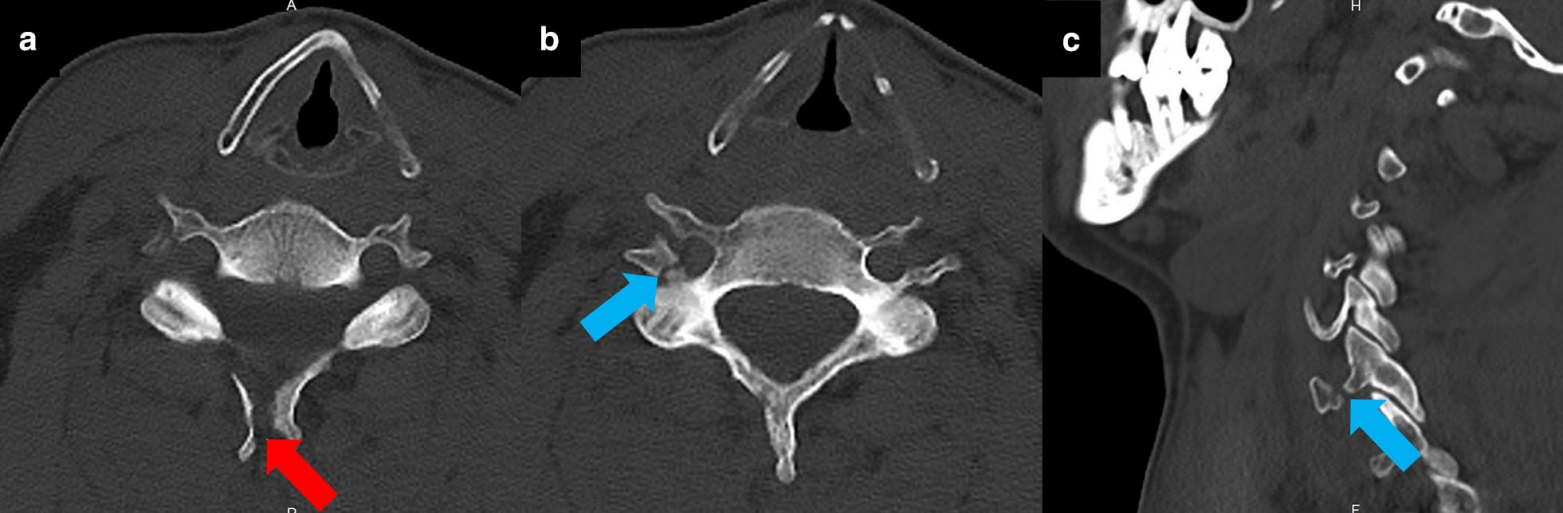
Diagnostic error. A 48-year-old male patient was admitted to the ER with head trauma after falling from a height of 2 m. The on-call radiologist reported a displaced fracture at the spinous process of the C6 vertebra (red arrow, **a**). Another displaced fracture at the posterior and lateral wall of the right transverse foramen of the C6 vertebra was missed (blue arrows, **b**, **c**). Emergency physicians were informed about the fractures after a second review of CT images. CT angiography performed to rule out vertebral artery injury, revealed no vascular injury

**Fig. 2:**
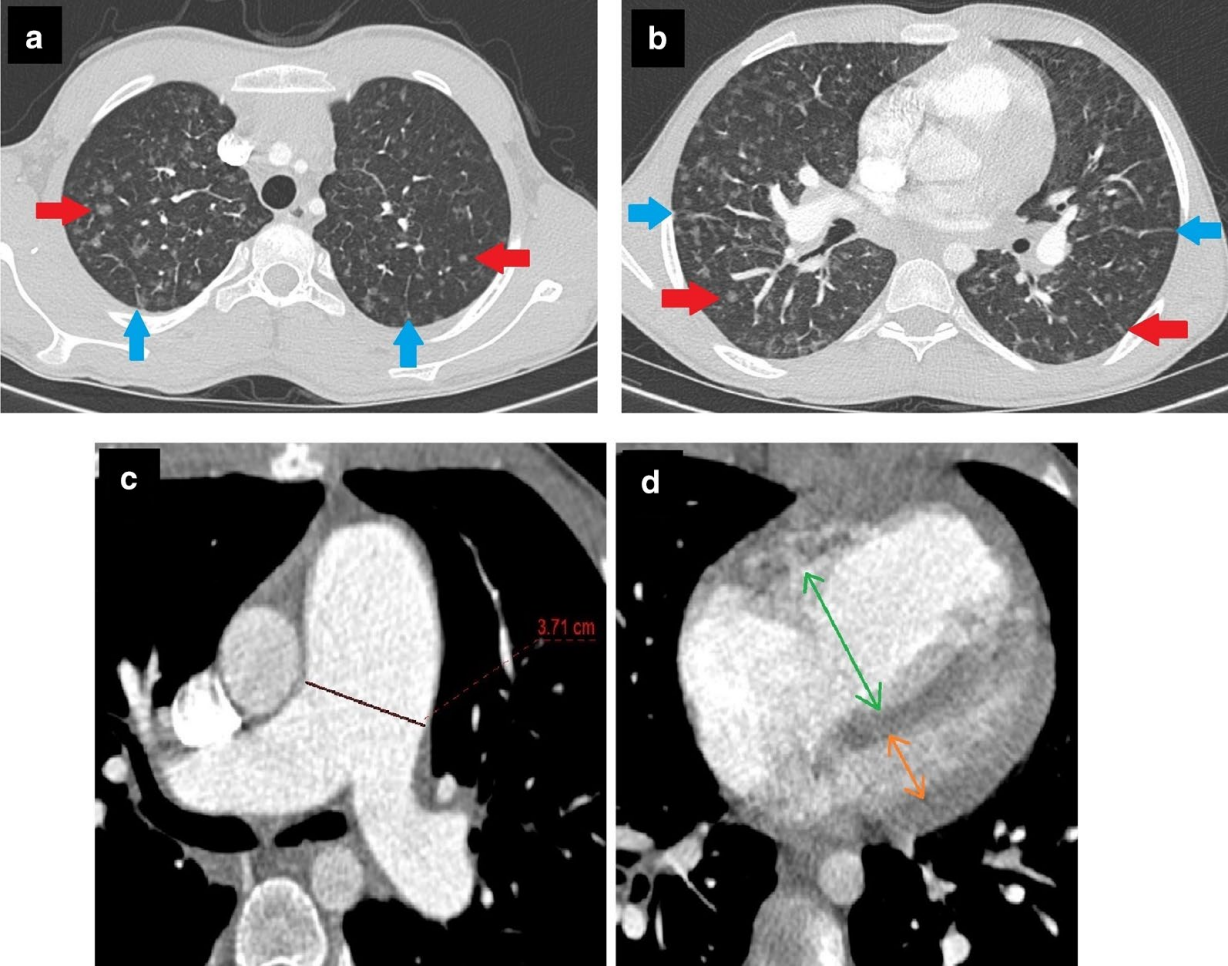
Discrepancy**.** A 13-year-old male patient with a history of pigeon-feeding presented at the Pediatric Department with the complaint of dyspnea. Clinicians suspected “hypersensitivity pneumonitis” as the first differential. The patient was hospitalized for further management. Serological tests proved the presence of antibodies against specific avian proteins. Thorax CT showed multiple ground-glass nodules (red arrows, **a**, **b**). A radiologist interpreted these nodules in favor of "hypersensitivity pneumonia" considering the clinician’s opinion, patient history and serological test results. According to another radiologist, pulmonary veno-occlusive disease was more likely, because of accompanying interlobular septal thickening (blue arrows, **a**, **b**), increased pulmonary artery diameter (**c**) and right ventricular dilatation (**d**). Although the radiologists were aware of each other’s opinions, both had plausible and supportive arguments for their provisional diagnosis. After a brief discussion, both possibilities were mentioned in the report, but both radiologists thought their own diagnosis was correct. After evaluating both possibilities in the multidisciplinary meeting, it was decided how to proceed with patient management

Diagnostic error is a condition that could harm the patient, with no acceptable cause and no scientific data for defense, approved by all experts in this field (Fig. 1). Discrepancy refers to a reasonable difference of opinion between radiologists about a finding or diagnosis. It differs from error because discrepancies can be justified based on a range of scientific data, such as clinical information, laboratory results or radiological patterns [3, 5] (Fig. 2).

### Common radiological error types

**Fig. 3:**
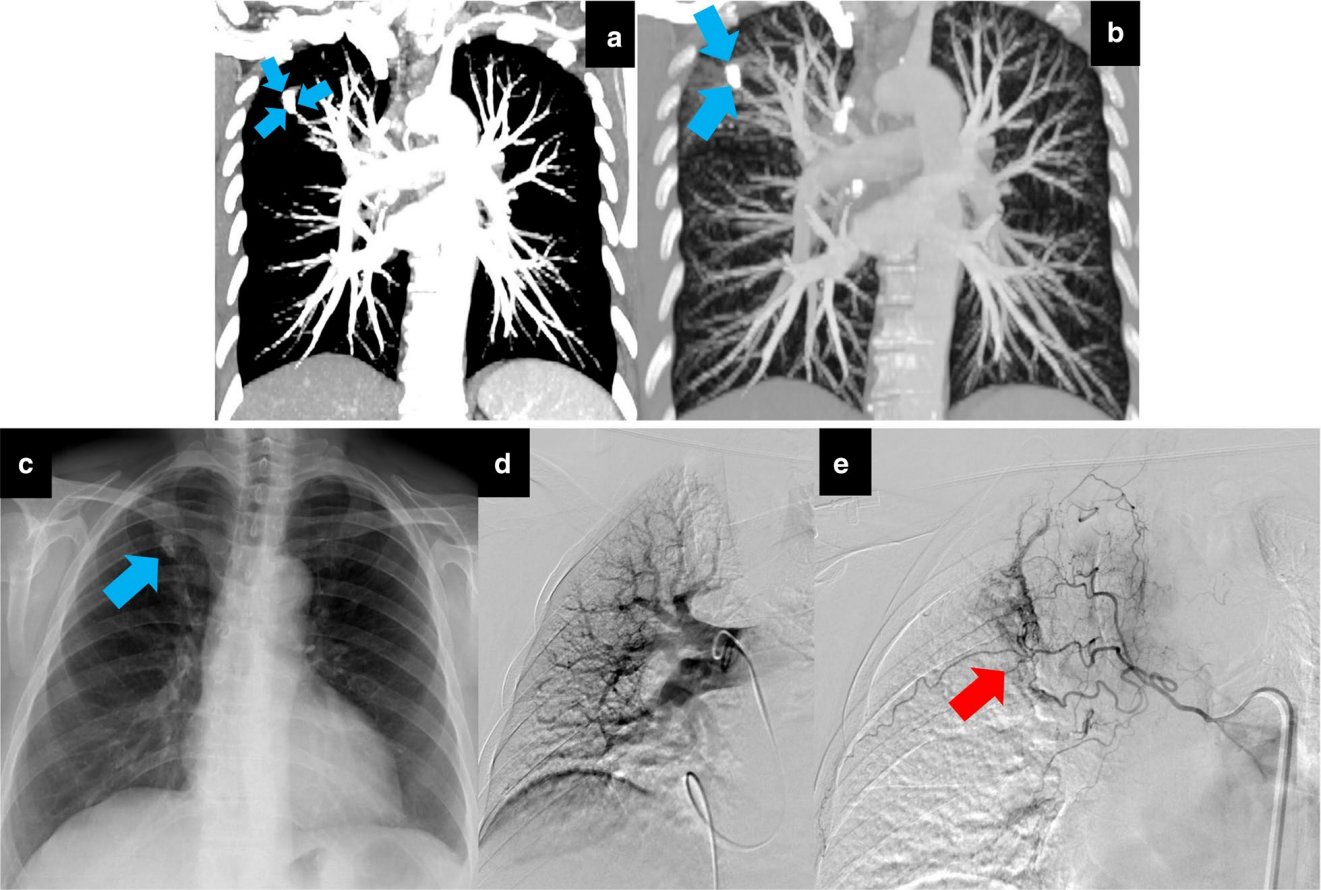
An over-reading (type 1) error with a positive effect on patient management**.** When evaluating contrast-enhanced CTs, failure to adjust the window settings may cause calcified lesions to be confused with vascular lesions (blue arrows, **a**, **b**). A 67-year-old female patient presented with the complaint of hemoptysis to another hospital. Before that admission, she had been investigated several times for hemoptysis in different medical centers via CTA, which had shown no apparent causes and no dilated bronchial artery. The patient had been discharged each time because of negative imaging findings and regression of symptoms. Upon this last admission, pulmonary CTA was performed and reported as «Consistent with pulmonary artery aneurysm in the right lung apex.». The patient was referred urgently to our vascular interventional unit. Invasive pulmonary angiography revealed nothing abnormal (**d**). It was later understood that a calcified nodule at the right apex (blue arrow, **c**) caused the over-reading error by mimicking an aneurysm. It was decided to perform bronchial arteriography following pulmonary angiography while the patient was already in the operating room because of the long-term recurrent hemoptysis history. Dilated, distorted and tortuous bronchial vessels detected on bronchial arteriography (red arrow, **e**) were embolized. Throughout a one-year follow-up period, the patient had no recurrence of hemoptysis. As shown in this case, errors do not always cause patient harm. Occasionally, an error may have a positive effect on patient management. The diagnosis and treatment process of the underlying cause of the patient’s recurrent hemoptysis was accelerated owing to an over-reading error causing an urgent referral to our angiography unit

**Fig. 4:**
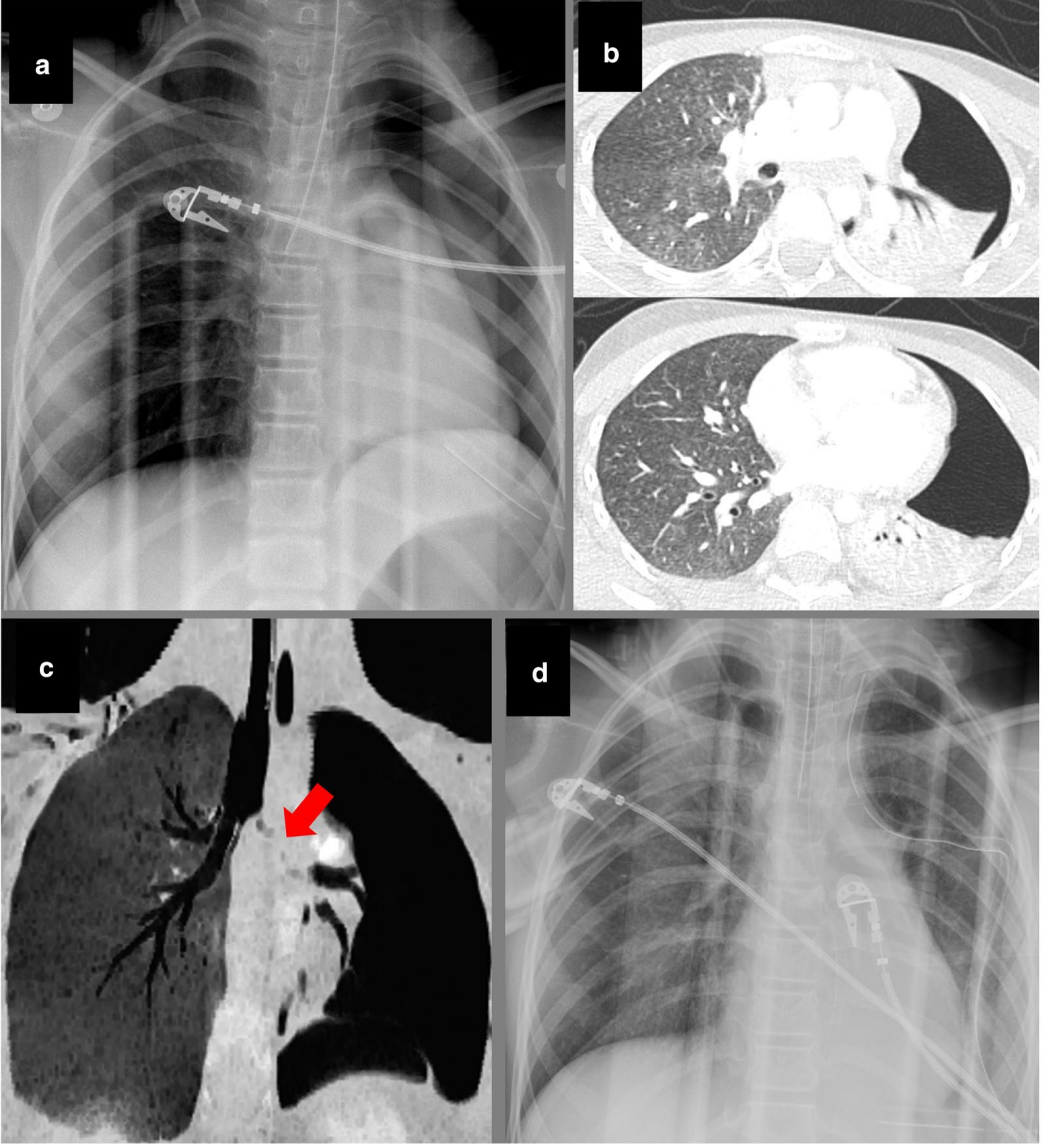
Faulty reasoning (type 2) error with anchoring and confirmation bias. A 10-year-old female patient was admitted to the ER after MVA. She had signs of severe head trauma and was intubated in the ER due to altered mental status. Following intubation, the absence of left lung ventilation was noticed, and a chest tube was placed in the left lung. Left-sided pneumothorax and misplaced intubation cannula extending to the bronchus intermedius were seen on the control chest radiograph (**a**). After stabilizing the vital signs, thorax and abdomen CTs were performed in the second hour of admission to assess the severity of the traumatic injury. Thorax CT showed “collapsed left lung with pneumothorax” (**b**, **c**) and “discontinuity of left main bronchus” (red arrow, **c**). The on-call radiologist evaluated those findings in favor of “bronchial rupture” and “fallen lung sign.” After making a preliminary report, the radiologist was called by a clinician, who informed him that patient’s intubation was traumatic and difficult, and asked him whether these findings could be secondary to “acute bronchial obstruction and subsequent collapse.” The radiologist rejected this possibility without thinking and stated that the findings he saw were compatible with bronchial rupture. Following the telephone call, the radiologist scanned the literature and read that bronchial rupture is usually seen with severe accompanying thoracic injuries and is associated with pneumomediastinum and pneumothorax. Although the radiologist knew that the patient did not have any other thoracic injury or pneumomediastinum, he sought exceptional cases that supported his pre-diagnosis, and he stuck with his first decision. The patient’s poor general condition did not allow surgical intervention or bronchoscopy. She was followed up with chest tube drainage and bronchial aspiration. On the second day after admission, portable chest radiography showed that the left lung ventilation was markedly normalized (**d**). When the findings were evaluated retrospectively, it was understood that bronchial rupture was unlikely, and the diagnosis was compatible with “acute lung collapse and pneumothorax ex vacuo secondary to the traumatic intubation.” Thus, the radiologist, under the influence of anchoring confirmation bias, had falsely attributed the findings he detected in favor of a false pathology

**Fig. 5:**
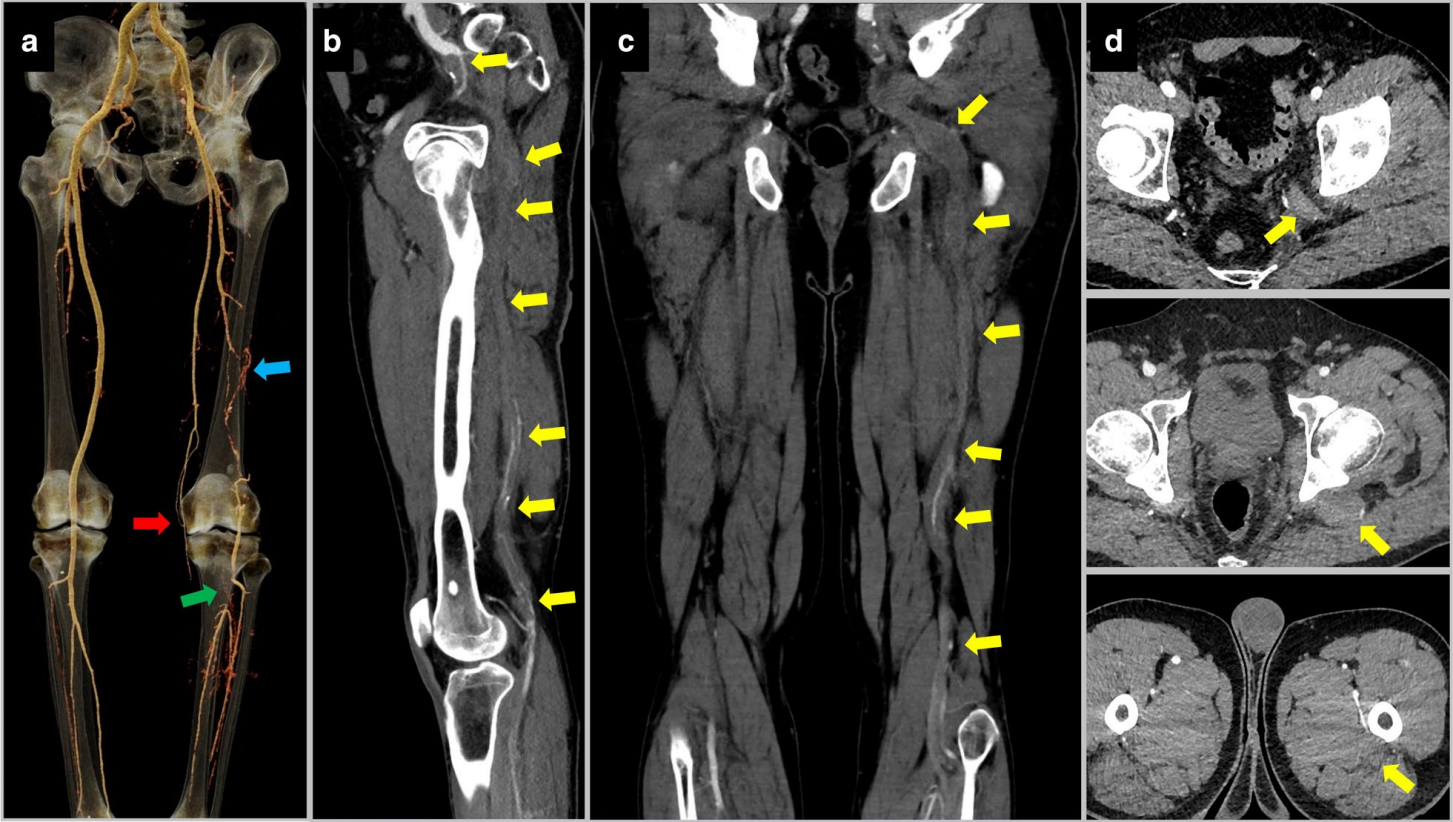
Lack of knowledge (type 3) error. A 55-year-old male patient underwent lower extremity CTA due to signs of peripheral artery disease in the left lower extremity. The radiology resident who examined the CTA for the preliminary results saw that the left superficial femoral artery was of thin caliber and ended at the knee level. He thought that it was chronically occluded (red arrow, **a**). He also thought that collaterals between the left deep femoral artery and popliteal artery (blue arrow, **a**) had developed secondary to the thrombi in the left popliteal artery (green arrow, **a**). When the radiologist referred the CTA to the attending physician, he learned that the patient’s left internal iliac artery continued as the popliteal artery, which is a variation called persistent sciatic artery. The persistent sciatic artery of the patient was thrombosed throughout its course (yellow arrows, **b**, **c**, **d**). The resident could not recognize this variation and the pathology due to the lack of knowledge. Although this is not a common variation that should be known by every radiologist, in this case, failure to recognize the thrombosed persistent sciatic artery could have adversely affected the possible interventional treatment plan

**Fig. 6:**
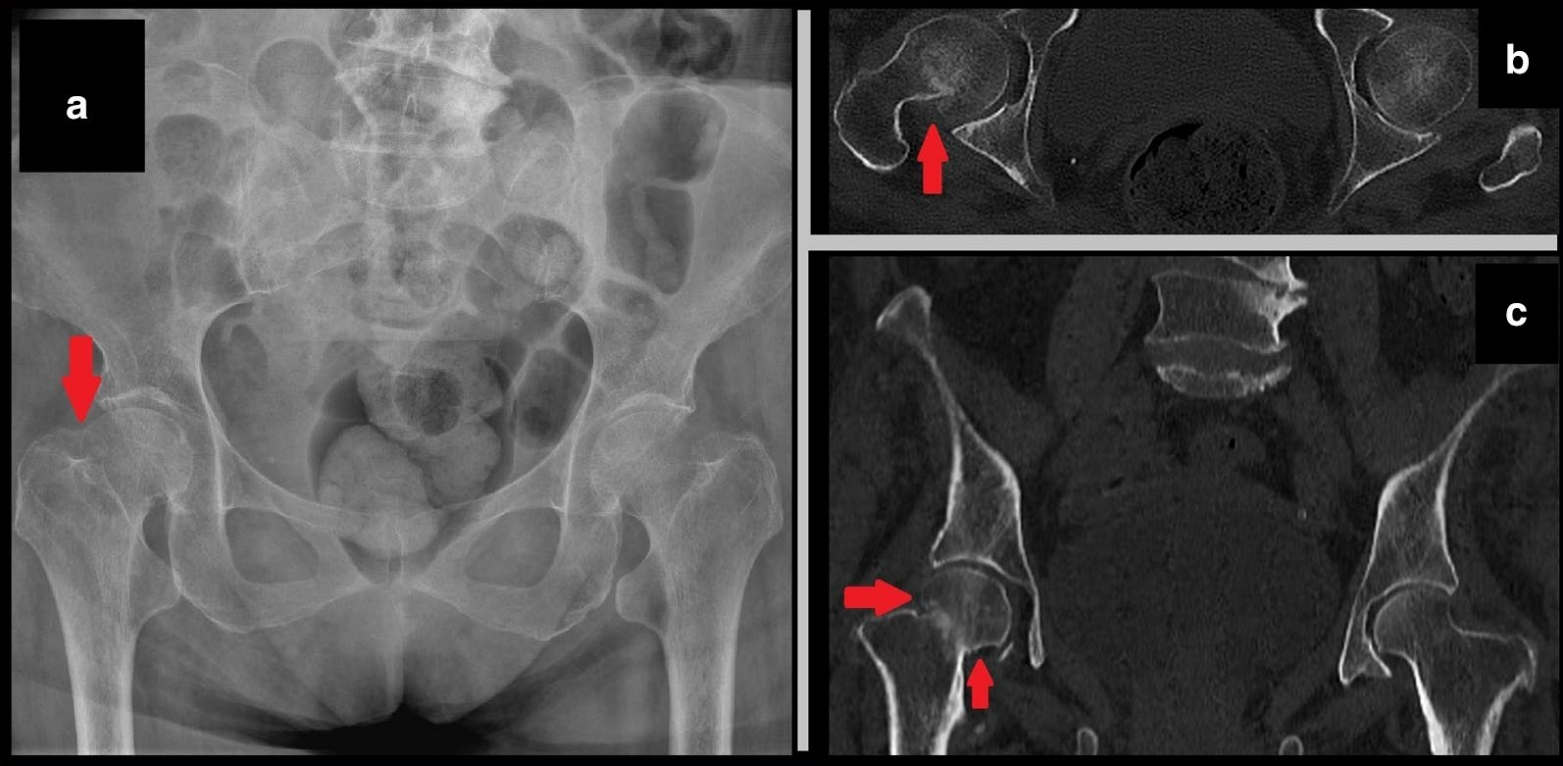
Under-reading (type 4) error with inattentional bias (Gorilla effect). A female patient was admitted to the emergency department with bilateral hip pain after falling down stairs. X-ray and pelvic CT were interpreted as “normal” by the on-call radiologist. However, shortening of the right femoral neck (red arrow, **a**) and a hyperdense line due to impacted fracture (red arrows, **b**, **c**) were missed. After retrospective detection of fracture, the radiologist was asked about the cause of missing these findings. He stated that he did not expect to see a hyperdense impaction fracture at all while evaluating that particular case. The prejudice of “fractures are seen as hypodense lines” caused inattentional blindness. A hyperdense fracture line is an uncommon finding, and due to its unexpected nature, may result in an under-reading error

**Fig. 7:**
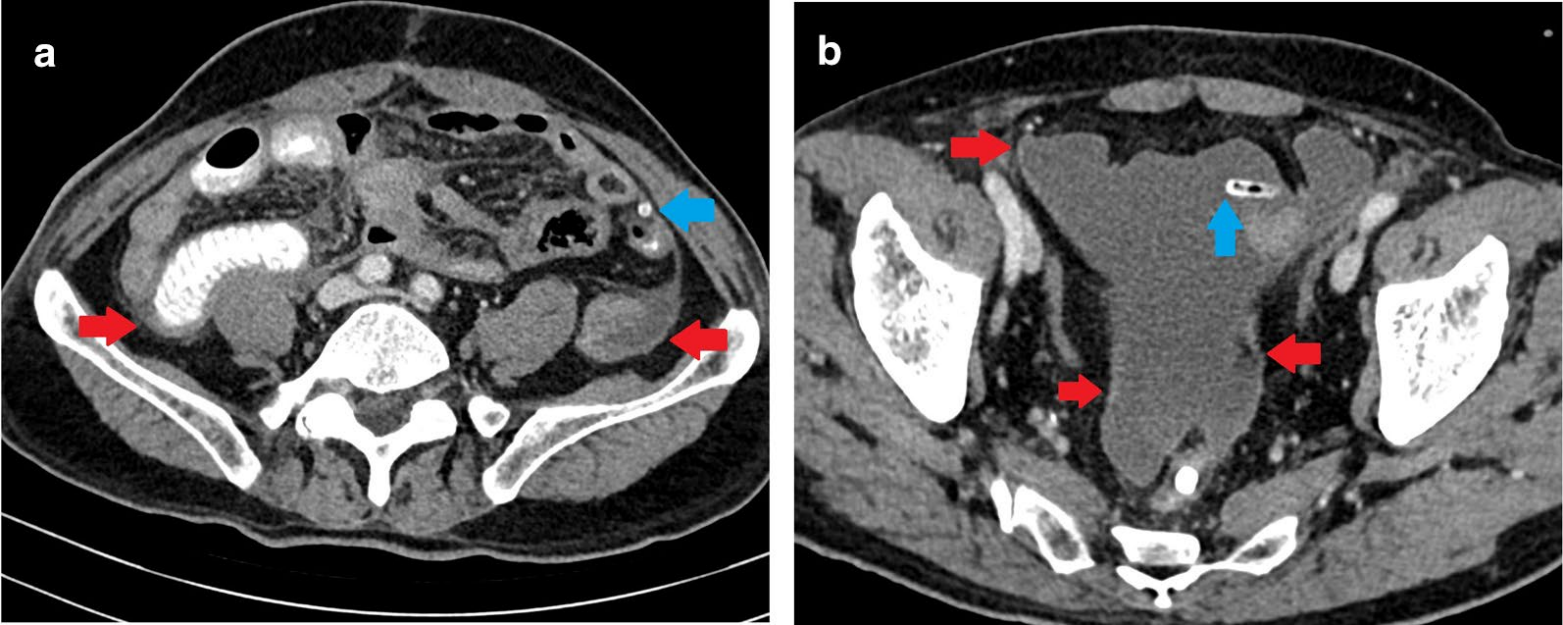
Premature closure bias causing an under-reading (type 4) error. A 52-year-old male patient with metastatic gastric cancer was brought to the emergency room due to abdominal pain, distension and discomfort. Abdominal CT showed thickened and enhanced peritoneal layers (red arrows **a**, **b**) and accompanying pelvic-free fluid/mesenteric congestion. The radiologist prematurely jumped to the conclusion of “peritoneal carcinomatosis” because of the cancer history and did not think about other reasons that could cause those imaging findings. In the following period, it was retrospectively understood that there was a displaced jejunostomy catheter (blue arrows **a**, **b**) into the peritoneal space. Due to premature closure bias, the radiologist did not pay enough attention to the rest of the examination and overlooked the displaced catheter. The patient was hospitalized and given long-term treatment. After removing the misplaced catheter and completing the appropriate treatment of peritonitis, induced by nutrients given through the jejunostomy, the patient’s complaints completely recovered

**Fig. 8:**
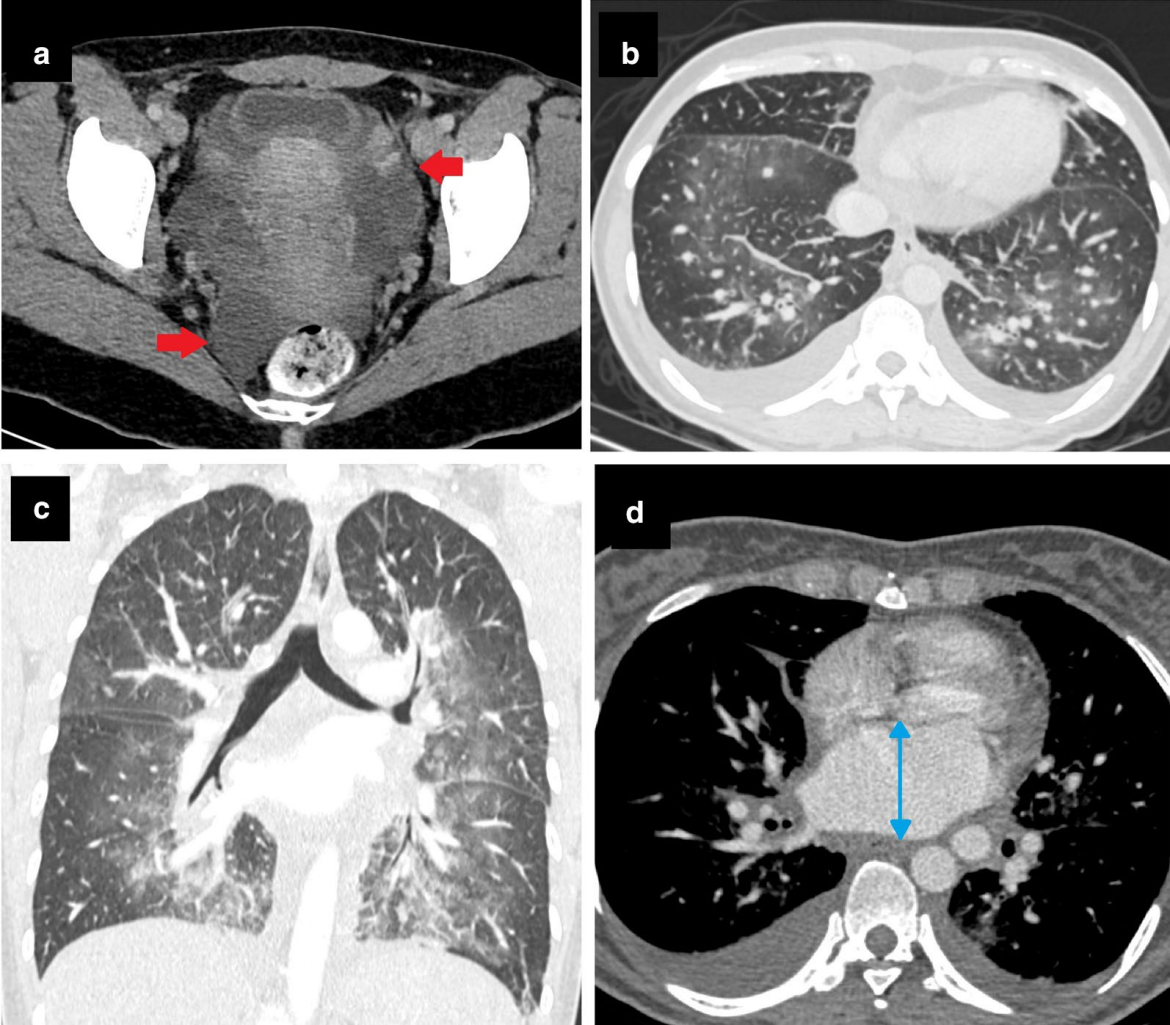
Poor communication (type 5) error with attribution bias. A 27-year-old female patient with no known disease presented at the Emergency Department with abdominal discomfort. After the first evaluation, an abdominal CECT was performed (**a**, **b**). Bilateral lung bases were also partially involved in abdominal CECT (**b**). CT was reported as “free fluid in the pelvis (red arrows **a**), bilateral pleural effusion, interlobular septal thickenings, and bilateral central ground-glass opacities in lung bases” (**b**). Although these are well-known findings of cardiac congestion, they were not attributed to cardiogenic edema because of the patient’s age and clinical history. Instead, findings were only described without any comment or impression in the report, and clinicians were expected to read the report and evaluate findings. Six hours later, the patient became dyspneic and tachypneic. Contrast-enhanced thorax CT (**c**, **d**) revealed findings compatible with pulmonary edema (**c**) and enlarged left atrium (blue double-sided arrow, **d**). Echocardiography, performed to rule out cardiac abnormality, showed severe mitral stenosis, possibly due to rheumatic heart disease. Attribution bias due to the “young age, clear medical history and irrelevant symptoms of the patient” caused the radiologist to avoid making any comments about the possibility of cardiac congestion in the report. As a result, poor communication between the radiologist and clinicians due to the lack of necessary interpretation in the report caused a delay in diagnosis

**Fig. 9:**
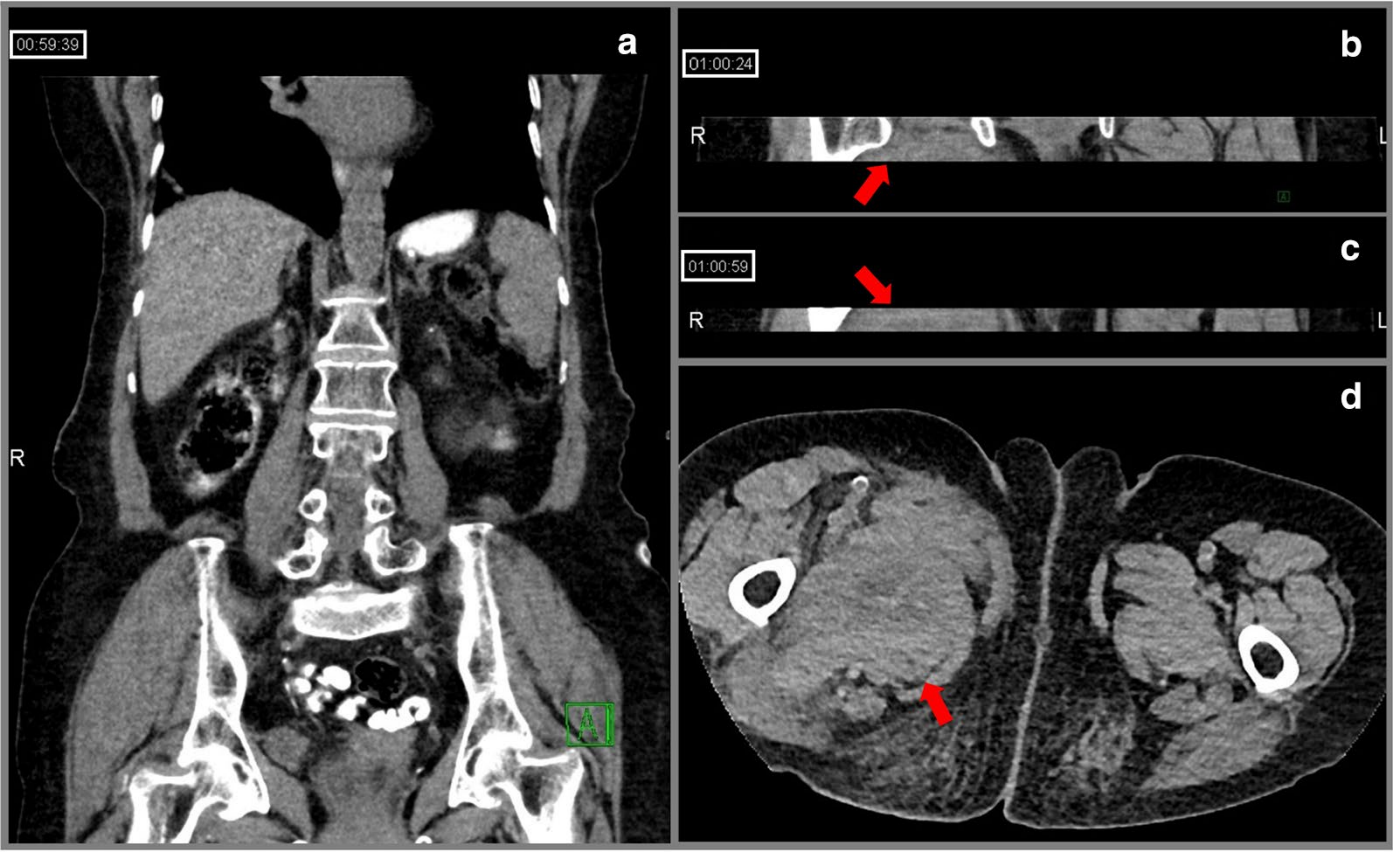
Technique-related (Type 6) error with poor communication. An 88-year-old female patient using an injectable anticoagulant was admitted to the emergency room with right lower quadrant pain. No finding to explain the symptom was observed on abdominal CT (**a**). Right inguinal swelling developed during follow-up and inguinal US revealed a large hematoma in this region. When the abdominal CT was evaluated retrospectively, it was noticed that, apart from the sections examined by the on-duty radiologists, two more series consisting of only 10–15 axial slices showing inguinal regions were separately uploaded to the PACS together with their coronal reformats (**b**, **c**). It was later learned that the CT technician had difficulty adjusting the field of view and had to take a few extra slices to cover the level of the symphysis pubis. However, he did not inform the on-call radiologists about this situation. Since the images were sent to the PACS piece by piece due to a technical error, and the radiologists were not informed about the problem due to lack of communication, the diagnosis of a clearly seen right inguinal hematoma was delayed (red arrows, **b**, **c**, **d**)

**Fig. 10:**
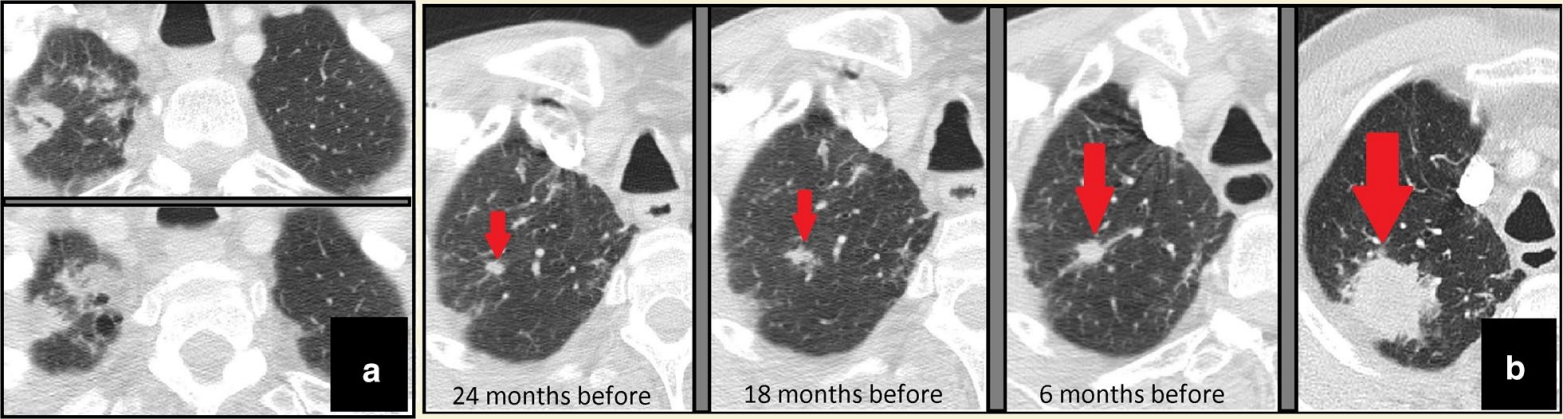
Prior examination (type 7) and satisfaction of report (type 12) errors. Follow-up images of a male patient with a history of larynx cancer showed that both lung apices were within the imaging field in all the control neck CTs. More prominently in the right apex, bilateral apical fibrotic changes were present and consistently reported (**a**). Due to reliance on previous reports, apical areas were not evaluated carefully. Consequently, a nodular lesion showing a gradual increase in size, located within right apical fibrotic changes, was repeatedly missed until it reached large dimensions (red arrows, **b**). Percutaneous biopsy and subsequent histopathological examination confirmed the diagnosis of primary lung adenocarcinoma

**Fig. 11:**
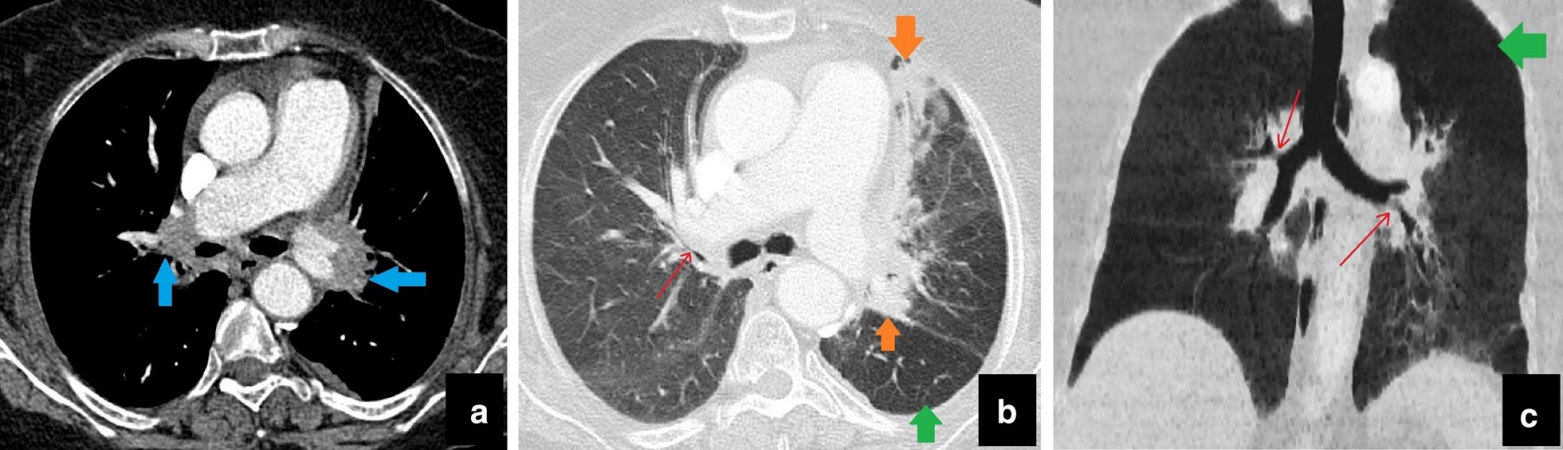
History-related (type 8) error. A 75-year-old female patient presented at the Chest Clinic with a progressive cough. Thorax CT was reported as "bilateral residual tumoral soft tissue (blue arrows, **a**) and parenchymal fibroatelectatic changes due to radiotherapy (orange arrows, **b**)." The referrer gave feedback to the radiologist about "the negative history of malignancy and radiotherapy," and intensive biomass exposure was mentioned. The CT was re-evaluated by the radiologist. Paramediastinal parenchymal changes (blue and orange arrows, **a**, **b**) were interpreted as a result of “bronchial anthracofibrosis” after combining the associated air trapping (green arrows, **b**, **c**) and bronchial narrowings (red arrows, **b**, **c**). Although the radiologist made an unwarranted assumption about prior malignancy and treatment, the main reason for this reporting error was insufficient clinical information about “biomass exposure.” This case is a good example of “error with no harm” thanks to the effective communication between the radiologist and the clinician via the clinician’s feedback

**Fig. 12:**
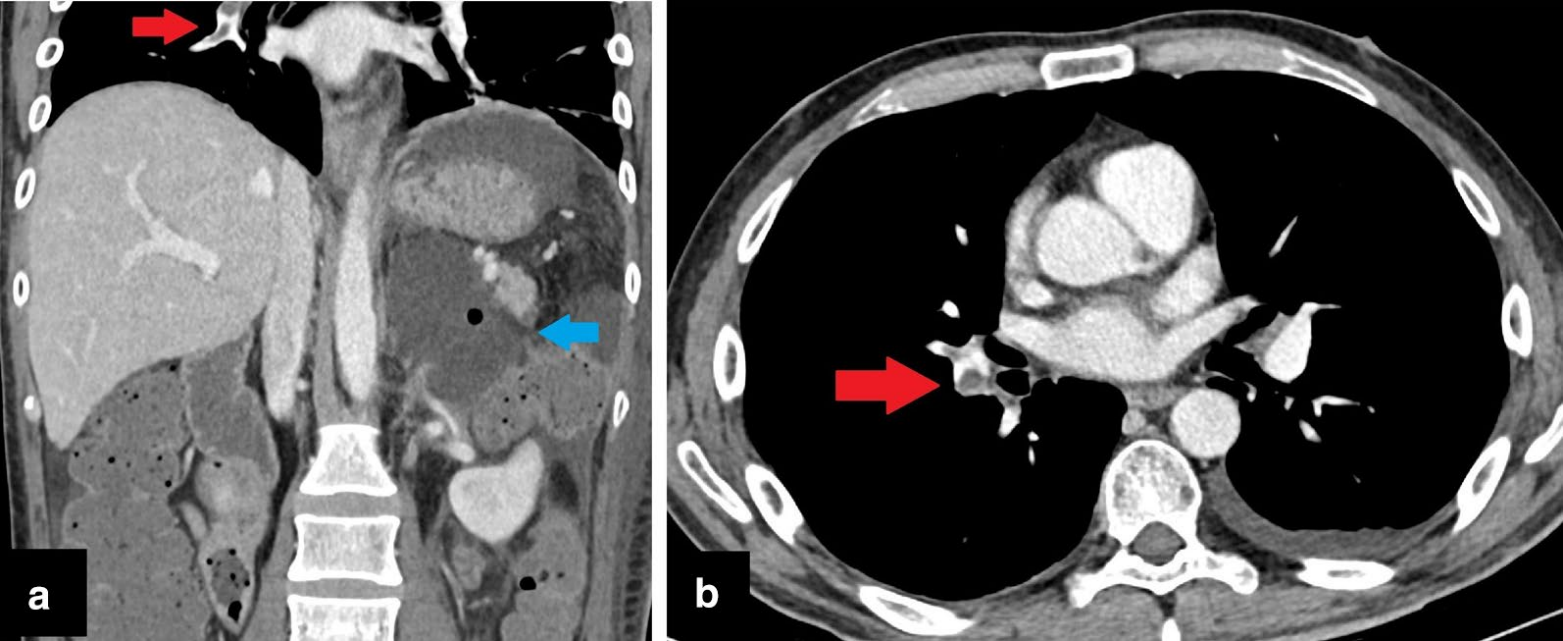
Location-related (type 9) error with under-reading. In a 28-year-old male patient with adrenalectomy, abdominal CECT was performed for evaluation of postoperative complications. Collection in the surgical area (blue arrow, **a**) and postoperative changes were reported. Although filling defects in the right pulmonary artery were involved in upper CT slices, they could not be noticed due to their locations (red arrows, **a**, **b**). As in this case, the marginal areas are prone to this type of error. There is also an accompanying under-reading error because the pulmonary emboli could be clearly seen and should have been reported

**Fig. 13:**
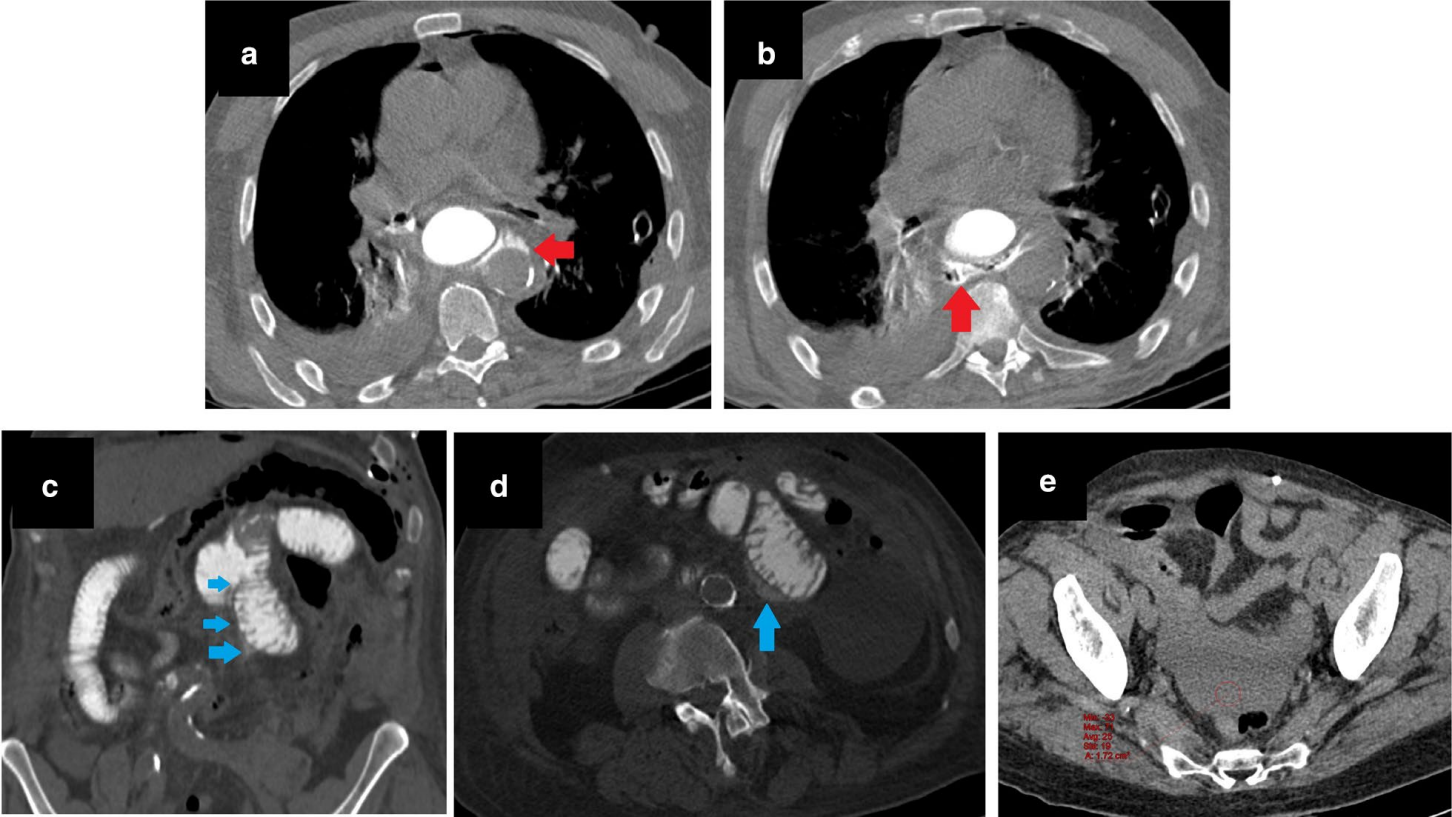
Satisfaction of search (type 10) error. A patient with gastric cardia tumor underwent total gastrectomy and distal esophagectomy. On the first postoperative day, dyspnea and abdominal pain developed. Anastomosis leakage was suspected, and thoracoabdominal CT with oral contrast agent was performed. The on-call radiologist noticed apparent contrast extravasation from esophagojejunostomy to the posterior mediastinum. The study was reported as “consistent with anastomosis leakage from esophagojejunostomy” (red arrows, **a**, **b**). Due to the “satisfaction of search” effect of this significant finding, the remainder of the examination was reported as normal. However, the patient’s abdominal complaints progressed, and the general condition of the patient deteriorated, so the patient was re-operated on, and additional jejunojejunostomy leakage was detected. When the preoperative CT was retrospectively evaluated, apparent signs of leakage in the jejunojejunostomy area were noticed. Linear hyperdensity consistent with leakage extending inferiorly from the anastomosis (blue arrows, **c**), extraluminal contrast material between jejunal loops (blue arrow, **d**), and pelvic fluid with relatively high density (red ROI mark, **e**) were overlooked findings

**Fig. 14:**
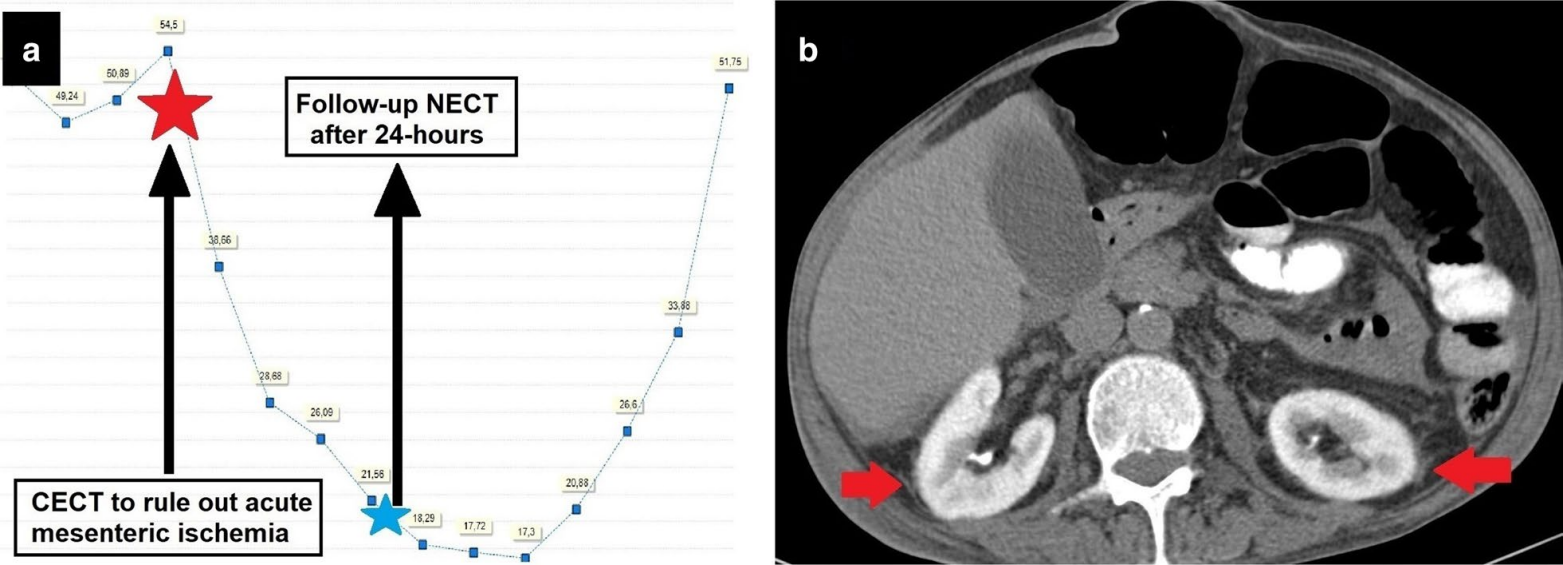
A complication developing under ideal conditions. A 64-year-old female patient with suspected sepsis was suffering from severe abdominal pain disproportionate to the clinical examination findings. Abdominal CECT was ordered to rule out “mesenteric vascular disease” after explaining all the risks of the procedure (including contrast-induced nephropathy) to the patient. Before the procedure, the glomerular filtration rate (GFR) was 54.5 ml/min/1.73 m2. After CECT, a progressive decline of GFR was observed. When the periprocedural GFR values were evaluated, it was clearly understood that contrast-induced nephropathy had developed (**a**). Follow-up NECT 24 h after the first examination revealed contrast retention of kidneys due to impaired filtration functions (red arrows, **b**)

**Fig. 15:**
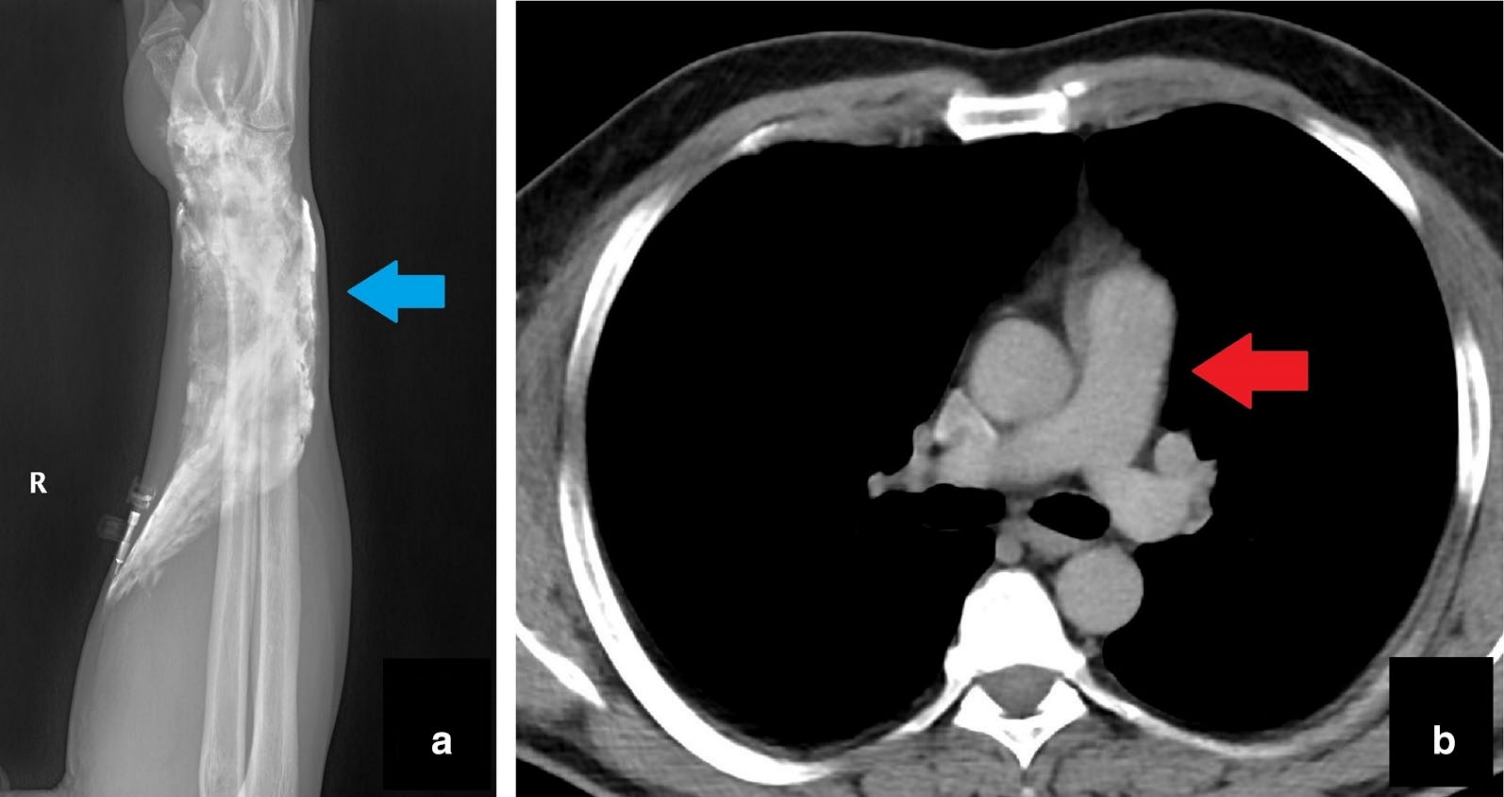
A complication (Type 11 error) causing acute harm to the patient and resulting in an insufficient examination. Thorax CT with pulmonary thromboembolism (PTE) protocol was performed for a dyspneic patient with a known breast cancer history. Just after the study, the patient complained of pain and swelling in the forearm. The X-ray of the upper extremity showed extravasated contrast agent to the wrist region (blue arrow, **a**). It was noted that the amount of intravascular contrast media was not sufficient for the evaluation of PTE (red arrow, **b**). Medical treatment was given, and the procedure was repeated. This case is a good example of “acute and temporary harm” caused by a periprocedural complication

Diagnostic errors constitute a large and complex issue that needs to be addressed, as they can prevent proper patient management, and a delayed diagnosis could lead to important consequences. Different classifications have been proposed at various times for the classification of diagnostic errors to facilitate their comprehensibility. The most broadly accepted classification was developed by Kim and Mansfield. According to this classification, diagnostic errors are examined in 12 groups based on the cause of the error [3, 4, 6, 7]:

1. *False-positive or over-reading error* In this scenario, an abnormality is noticed during the radiological examination. However, this finding is given more clinical value than it deserves and may consequently cause unnecessary diagnostic/therapeutic effort (Fig. 3) [6, 7].
2. *Faulty-reasoning error* In this type of error, detected abnormal radiological findings are thought to be associated with a false clinical entity, mostly due to cognitive biases such as hindsight bias or attribution bias (Fig. 4) [6–8].
3. *Lack of knowledge error* This error type occurs when a pathological finding is noticed but cannot be interpreted correctly due to lack of adequate knowledge or experience of the radiologist about the finding, despite the availability of adequate clinical information (Fig. 5) [3, 6–8].
4. *Under-reading error* This is the most common error type, in which an examination is reported as normal, although there is an undeniable and detectable abnormal finding. (Figs. 6, 7) [3, 6, 7].
5. *Poor communication-related error* In this scenario, the abnormal finding is recognized and accurately reported. However, the diagnostic message is not delivered to the clinician because of communication-related problems. In some circumstances, referrers may not be aware of the importance of the reported findings in the radiology report due to individual failings in communication between radiologist and clinician or systemic issues such as lack of multidisciplinary meetings and teamwork, increased workload and understaffing. Poor communication errors may also result from referrers not understanding radiology reports, lack of knowledge about the meaning of radiological findings or their own biases. Last but not least, typing errors in the report may disrupt the communication between the radiologist and the referrer, irrespective of the quality of the information provided by radiologists or the competence of referrers (Fig. 8, 9) (Table 1) [3, 6–8].
6. *Technique-related error* This error occurs because of low technical quality, inaccuracies during the image acquisition process or selection of the wrong technique or modality, resulting in a decreased possibility of detecting abnormal findings and sometimes makes diagnosis impossible (Fig. 9) [1, 6, 7]
7. *Prior examination-related error* The underlying cause of this error is skipping the “comparison with previous exams” step, which is indispensable during radiological evaluation. Each evaluation must be compared with previous examinations to increase the likelihood of detecting pathological findings. However, when doing this, one must be careful about the ‘satisfaction of report’ error that will be explained later (Fig. 10) [6, 7].
8. *History-related error* This error includes inaccurate reporting faults when the radiologist is not provided with adequate or correct information about the patient’s clinical history (Fig. 11) [3, 6–8].
9. *Location-related error* Location-related error is characterized by the inability to recognize the pathological finding, which is seen within examination limits but falls outside the purposefully examined area, especially at the edges of the evaluated area (Fig. 12).
10. *Satisfaction of search* When the radiologist defines a pathological finding during the evaluation, other findings may be overlooked due to the satisfaction of the assessment and loss of motivation. Thus, accompanying findings may be under-read even if they are seen very clearly (Fig. 13) [6, 7].
11. *Complication* As a general description, complications are the conditions that occur during or after the procedures and are directly related to the nature of the procedure. They are unanticipated occurrences and may happen even under ideal conditions, making it controversial to define them as errors (Fig. 14) [6–8]. However, according to the Kim-Mansfield classification, the term "complication," as an error type, refers to an adverse event related to invasive radiological procedures (Fig. 15) [6].
12. *Satisfaction of report* This results from having undue confidence in the patient’s prior reports. As a result, if a wrong assessment has been made in the previous report, it will be repeated. This type of error is closely related to alliterative bias (Fig. 10) [6, 7].

### Underlying bias types of radiological errors

**Fig. 16:**
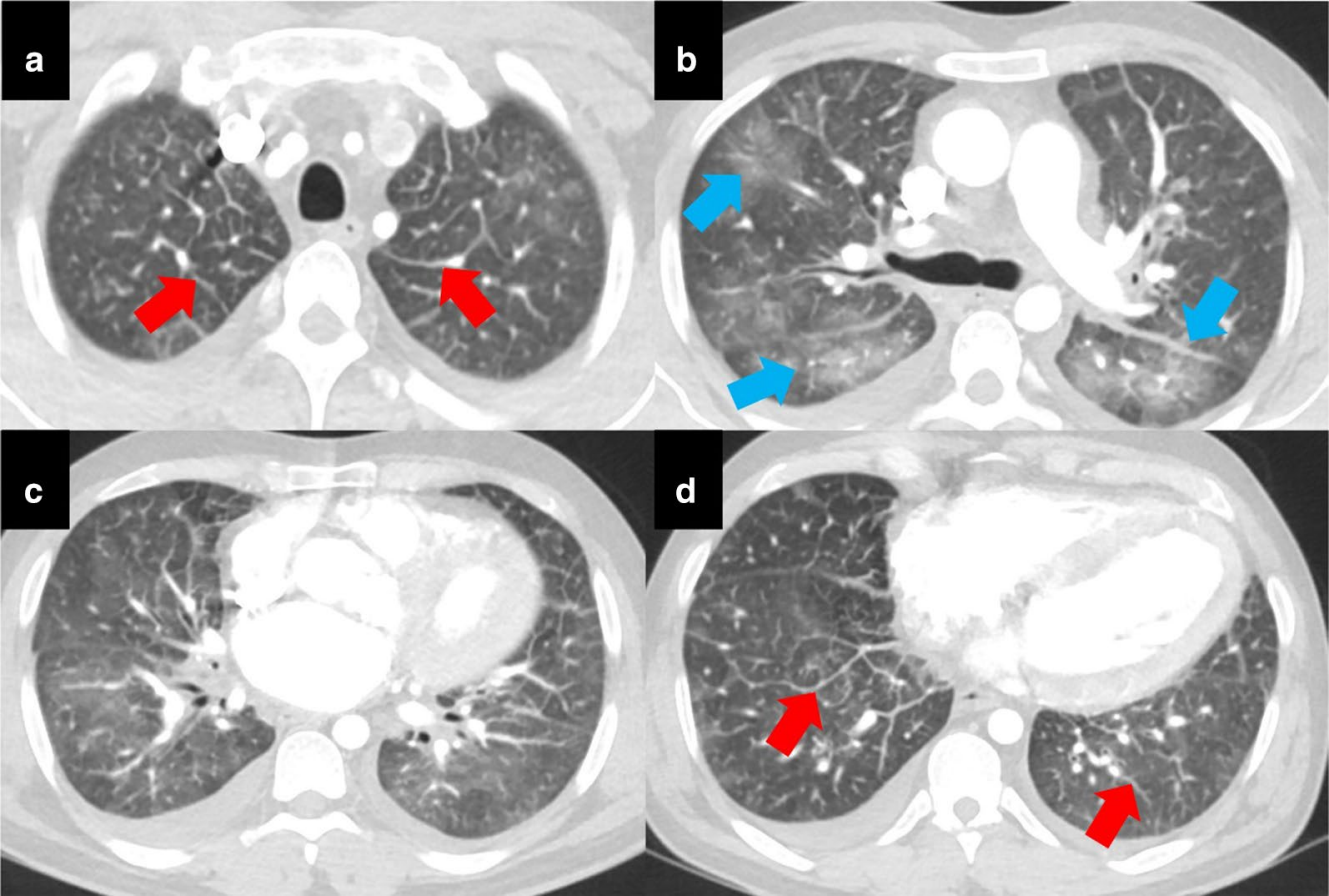
Availability and regret bias causing faulty reasoning error. A 41-year-old male patient with aortic valve stenosis was admitted to the Emergency Department with epigastric pain during the COVID-19 pandemic. A thoracoabdominal CT was ordered to assess the aortic stenosis, lung parenchyma and possible causes of epigastric pain. The on-call radiologist on the night-shift correctly described the CT findings as cardiomegaly, bilateral ground-glass opacities predominantly in the upper lobes of the lungs (blue arrows, **b**), thickening of the interlobular septae (red arrows, **a**, **d**) and bilateral pleural effusion. Before the CT of this patient, the radiologist had reported a large number of CTs compatible with viral pneumonia during the night-shift. In addition, he had overlooked a patient with positive chest CT finding for COVID-19 pneumonia on a previous night-shift, so he was overly sensitive about the diagnosis of COVID-19. Having been “burned once,” he did not want to underdiagnose the possibility of COVID-19. Therefore, these CT findings were attributed to pulmonary infection by indicating that COVID-19 pneumonia could not be excluded in the differential diagnosis. However, he did not mention the possibility of pulmonary edema. Viral pneumonia treatment was started due to the radiologist's opinion, and a reverse transcriptase polymerase chain reaction (RT-PCR) test for COVID-19 was ordered and reported as negative. On the following day, the attending emergency radiologist informed emergency physicians that the CT findings were primarily suggestive of cardiogenic pulmonary edema. The patient then showed a dramatic response to cardiogenic pulmonary edema treatment. As the frequency of viral pneumonia was greatly increased during the pandemic, and the radiologist had a lot of experience about this condition, availability bias resulted in misjudgment and faulty reasoning error. Although regret bias due to previous negative experience eliminated the possibility of overlooking another case of COVID-19 pneumonia for the radiologist, it also contributed to the error by affecting the thought process

**Fig. 17:**
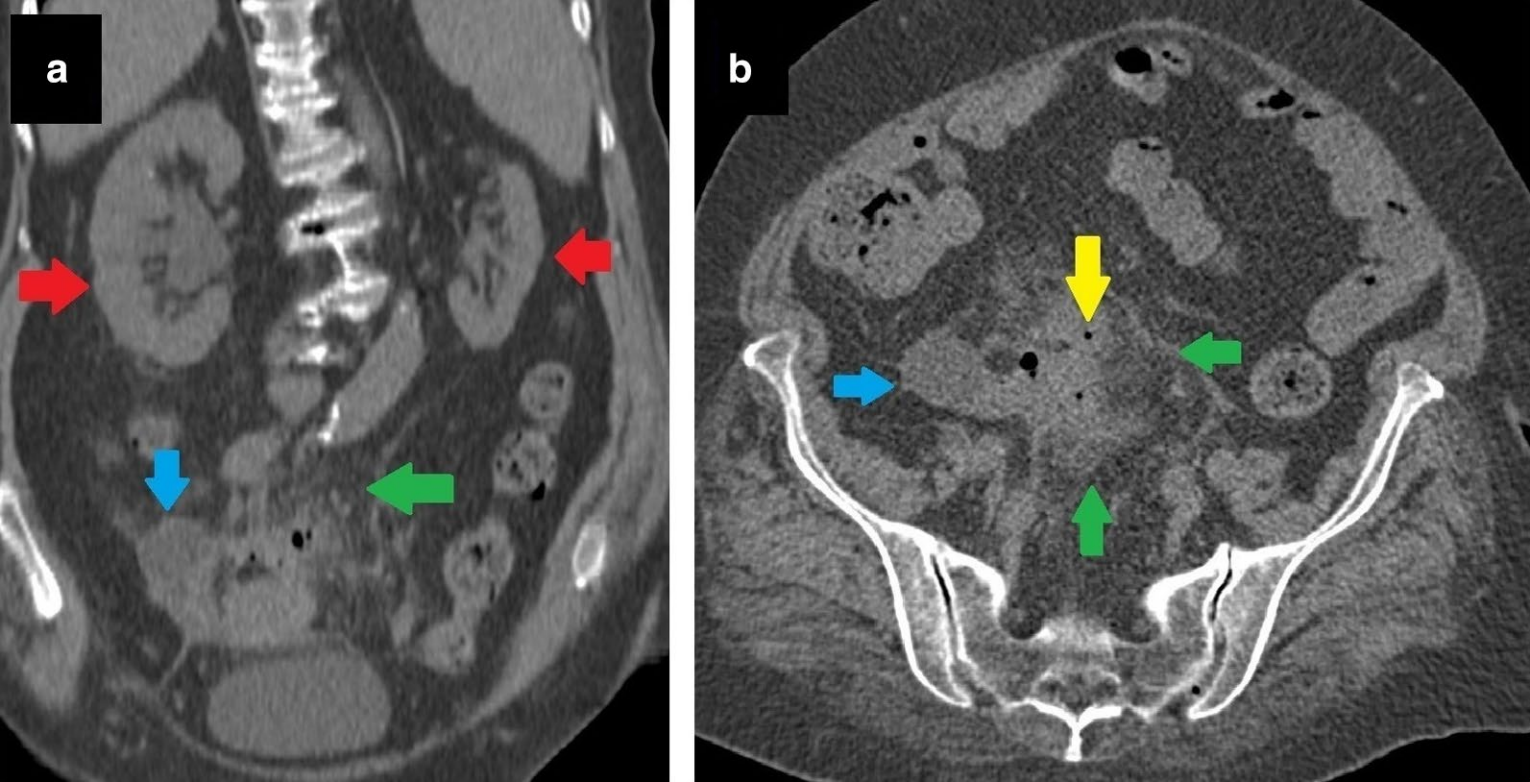
Framing bias causing an under-reading error. A 77-year-old female patient with a history of renal stone and recurrent urinary tract infections presented with lower abdominal pain. The patient’s symptoms did not regress under proper antibiotherapy. Non-enhanced CT was ordered for “evaluation of renal stones and urinary pathologies.” The CT was reported as “normal, except left atrophic kidney (red arrows, **a**).” However, a collection (blue arrows, **a**, **b**), free-air bubbles (yellow arrow, **b**) and mesenteric fat stranding (green arrows, **a**, **b**) adjacent to the sigmoid colon were overlooked due to the framing of clinical information. Although non-enhanced CT was technically inadequate to evaluate bowel pathologies, inflammatory changes were clearly visible on CT, and at least a suspicion should have been expressed. During follow-up, lower abdominal MRI revealed the final diagnosis as “perforated sigmoid colon malignancy” (not shown)

**Fig. 18:**
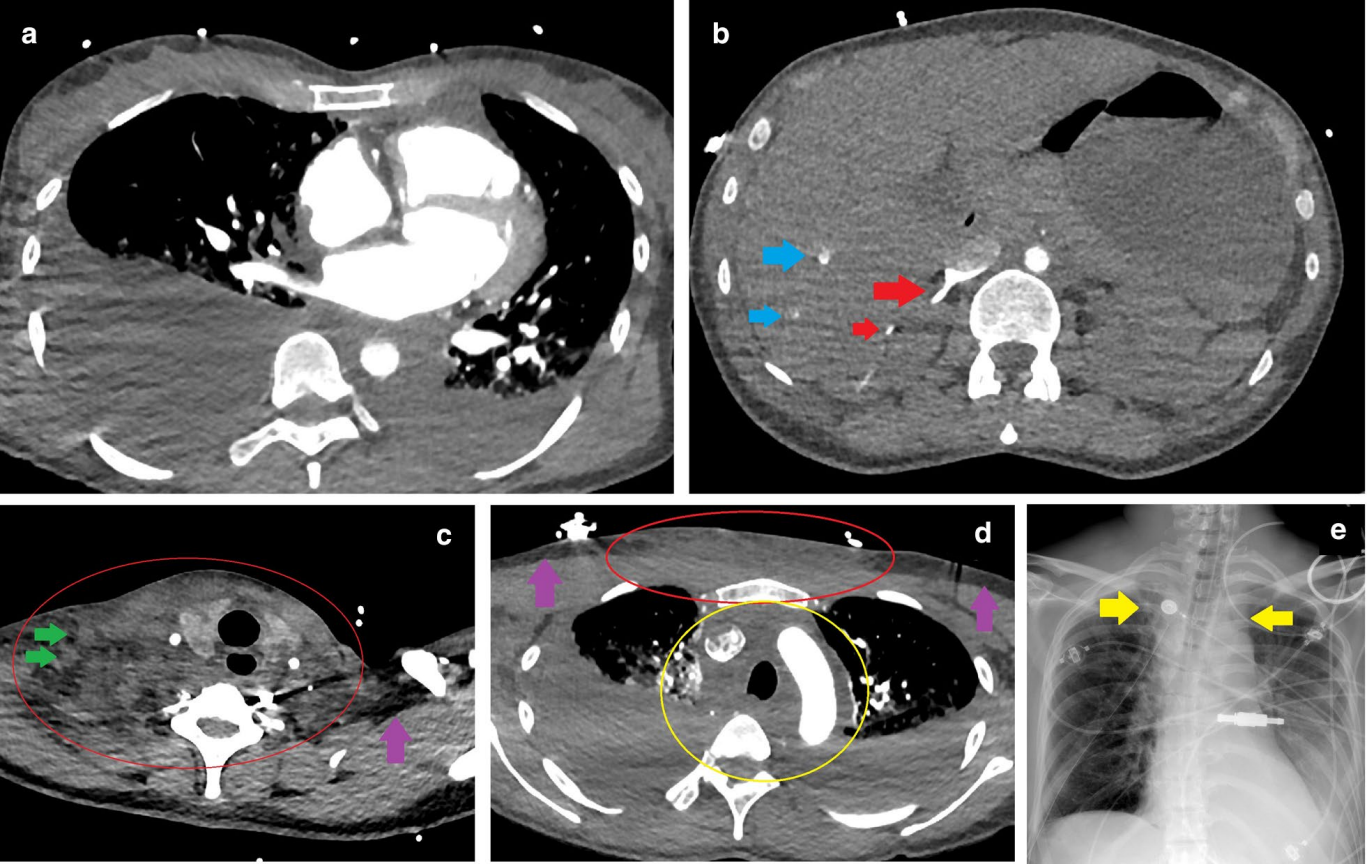
Hindsight bias. A 40-year-old female patient with no known disease history presented at the ER with complaints of chest pain, dyspnea and confusion. As severe back and neck pain had been ongoing for two days, aortic dissection was suspected. Triple rule-out CT showed bilateral massive pleural effusion (**a**, **d**) and severe contrast reflux into the inferior vena cava, right renal vein (red arrows, **b**), and peripheral branches of hepatic veins (blue arrows, **b**). These findings were suggestive of hemodynamic compromise. Pleural effusions were drained, and a significant amount of pus was observed. The patient was accepted as septic, and proper treatment was started. Microbiological evaluation of the pleural fluid was consistent with Streptococcus pyogenes, an unexpected pathogen bacteria in pleural samples. After feedback from the microbiologist, an ecchymotic area spreading from the neck to the anterior chest wall was noticed. Soft tissue infection was suspected. When the CT was retrospectively re-evaluated, it was understood that increased densities in subcutaneous fat tissues of the neck and anterior chest wall (red circles **c**, **d**) were not noticed by the on-call radiologist ( purple arrows **c**, **d**: normal fat tissue as a reference). Lymphadenopathies within the affected areas (green arrows, **c**), and obliteration of upper mediastinal fat tissues (yellow circle, **d**) were other considerable findings. Moreover, mediastinal widening suggesting mediastinitis was detected retrospectively on the chest X-ray obtained after thoracentesis (yellow arrows, **e**). Subsequently, the clinical situation rapidly deteriorated, the patient became hypotensive and a sharp myoglobin increase suggesting myonecrosis was observed. Acute kidney failure, acute respiratory distress and profound thrombocytopenia developed. After evaluating the clinical, radiological and microbiological findings altogether, the diagnosis of “streptococcal toxic shock” was made. The patient died three days after the onset of the complaints and one day after ER admission. With the knowledge of S.pyogenes presence in the pleura, history of neck pain and location of the ecchymotic area, it seems impossible to have overlooked changes in soft tissues and the upper mediastinum during retrospective evaluation (hindsight bias)

**Fig. 19:**
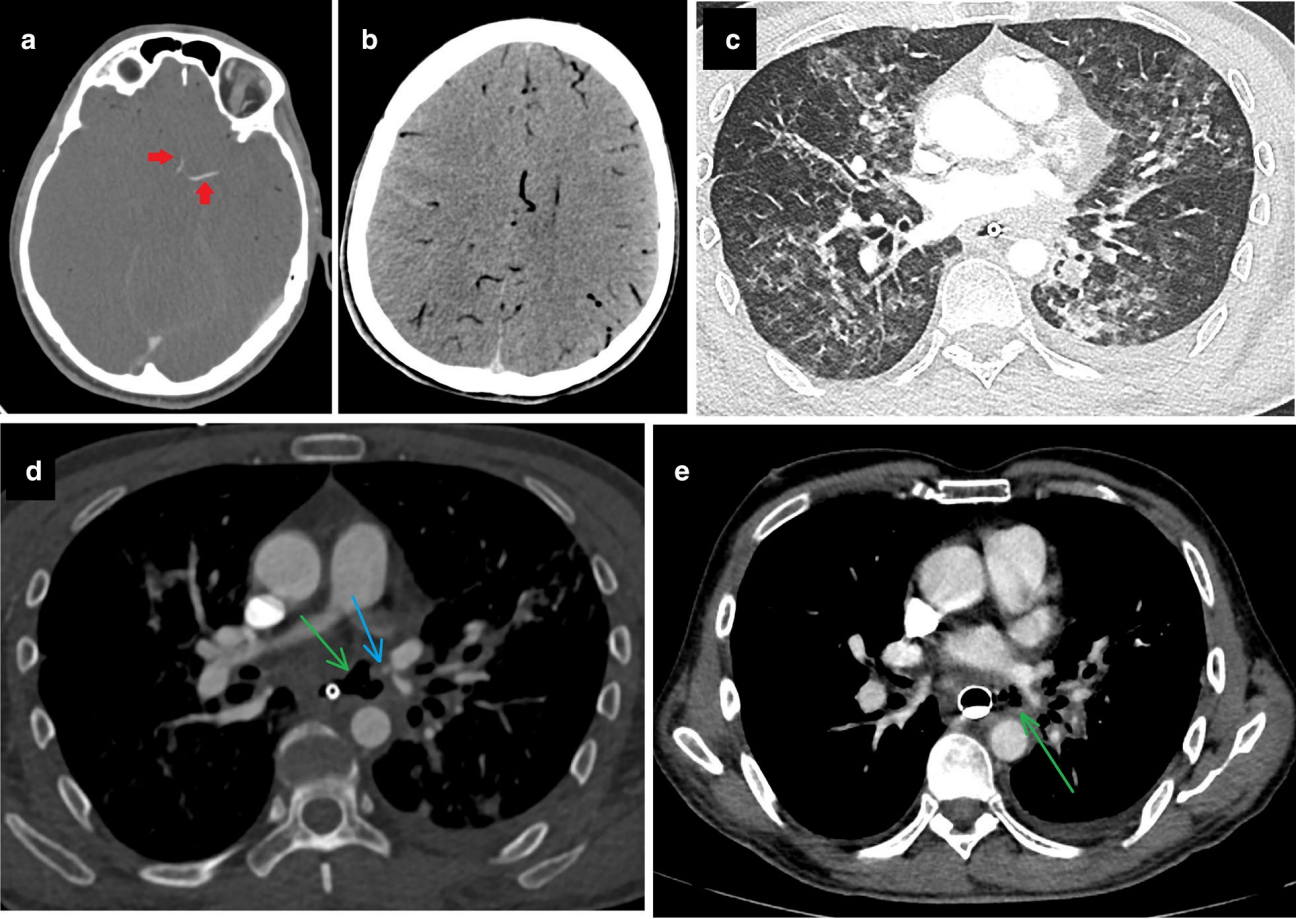
Zebra retreat and hindsight bias. A 75-year-old male patient with known tracheoesophageal fistula (green arrows, **d**, **e**) suddenly lost consciousness. Brain CT and CT angiography revealed edematous brain tissue, diffuse air within brain vasculature (**b**), and contrast filling limited to just proximal segments of the left middle cerebral artery and anterior cerebral artery (red arrows, **a**). Recent studies of the patient were re-evaluated to detect the etiology of cerebral air embolism. On thorax CT taken 3 days previously for hemoptysis, there was seen to be parenchymal ground glass opacities suggesting hemorrhage (**c**). In the mediastinal window, next to the fistula, there was an outpouching of the pulmonary vein (blue arrow, **d**) that had not been present on the previous study (**e**). The on-call radiologist had also noticed this newly developed outpouching but did not feel confident enough to mention this finding in the report due to the absence of apparent contrast extravasation into the tracheal lumen. Moreover, the radiologist had thought that hemoptysis originating from a pulmonary vein was quite an unexpected situation. This phenomenon is called a “zebra retreat.” It seems to be impossible to overlook or underrate that outpouching with “a priori knowledge of diffuse cerebral air embolism” due to the hindsight bias during retrospective evaluation

**Fig. 20:**
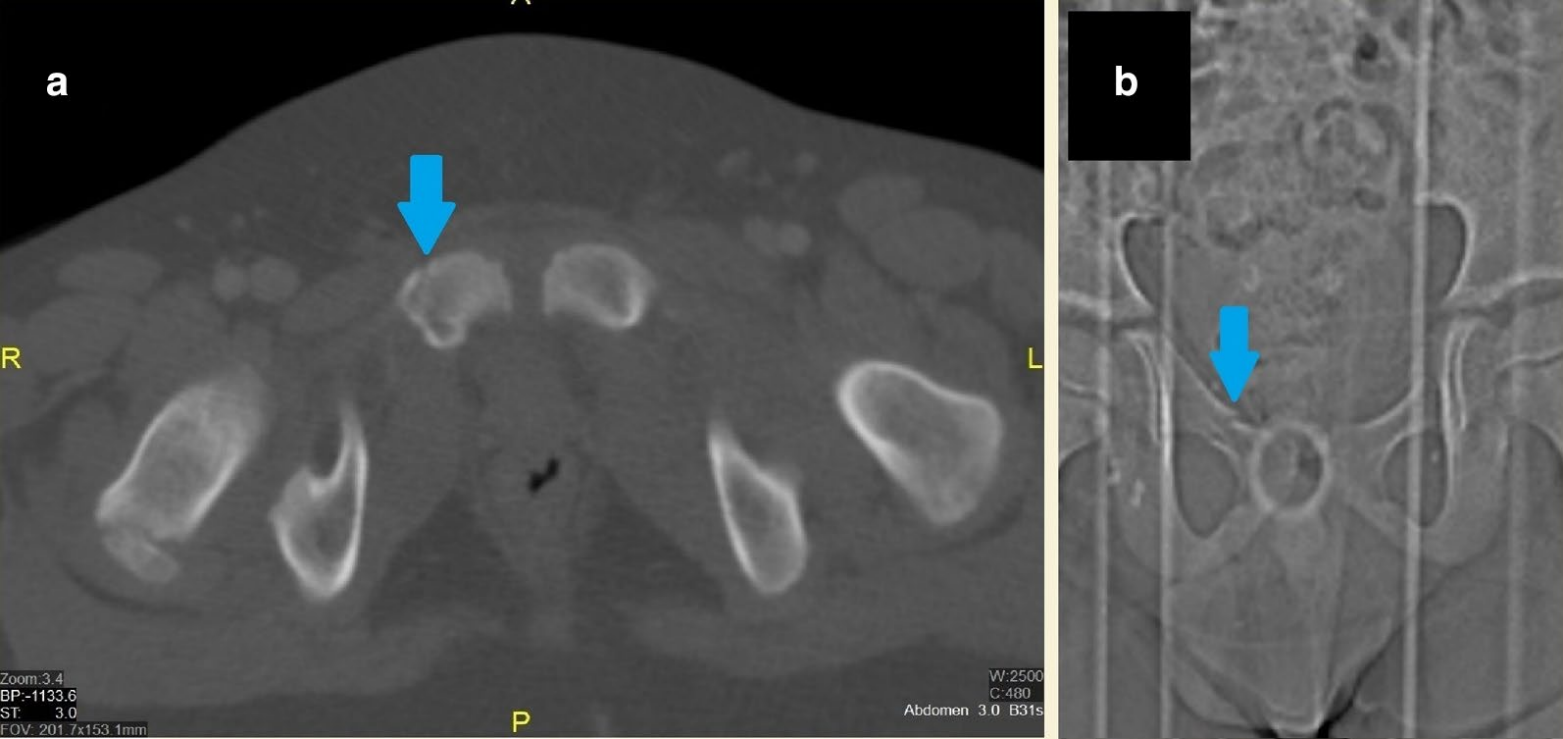
Scout neglect bias. An abdominal CECT of a male patient was performed to evaluate solid organ injury after a motor vehicle accident. A right pubic fracture (blue arrows) was masked in routine abdominal CT images of 3 mm slice thickness with a soft filter (**a**). However, it was easily seen in the scout image without any doubt (**b**)

Different biases are defined in the literature, which may affect the decision-making process when evaluating a radiological study. Biases may result in misinterpretation and different types of diagnostic errors, so awareness of certain types of biases can contribute to diagnostic accuracy. Biases can be classified as listed below [1, 3–6, 9]:

1. *Attribution bias *This is the tendency to attribute findings of a clinical condition by looking at specific characteristics of the patient; in other words, stereotyping (Fig. 8) [6, 7].
2. *Alliterative bias (Satisfaction of report) *Alliterative bias occurs when previous interpretations of a study influence the decision-making process of the current study (Fig. 10) [2, 4, 5].
3. *Availability bias *This type of bias is characterized by an increased tendency to make the decision under the influence of recently seen cases (Fig. 16) [1, 3–6, 9].
4. *Regret bias* This happens when radiologists worry about underdiagnosing a possibility and thereby over-report it due to fear of missing a diagnosis (Fig. 16) [7, 9].
5. *Framing bias* This bias type reflects the restriction of imaging assessment to the referral situation and clinical presentation framework (Fig. 17) [1, 4].
6. *Premature closure* This results from accepting an initial impression as a final diagnosis without any verification. Radiologists may be cognitively satisfied after discovering the first finding and end the search. Therefore, premature closure bias may overlap with the “satisfaction of search” error (Fig. 7) [3, 9].
7. *Inattentional bias *Inattentional bias, also named tunnel vision, may result in a diagnostic error due to an unusual appearance or location of findings (Fig. 6). This is also named the "Gorilla effect" after the famous study, in which a gorilla was inserted in a thorax CT by researchers and was overlooked by 83% of radiologists who were busy searching for lung nodules [10]. Inattentinal bias may cause devastating complications, even in cases with obvious imaging findings [3, 5, 9].
8. *Hindsight bias *This type of bias is characterized by believing that it would be easy to reach a correct diagnosis by ignoring or de-emphasizing the difficulties in making the initial diagnosis and discounting the scenario under which the decision making occurred. When specialists evaluate the findings retrospectively, they may have access to contributory information not available to the original reporters, and they tend to underestimate the difficulty of making an accurate diagnosis due to hindsight bias. In contrast to other types of bias, hindsight bias is retrospective in nature (Figs. 18, 19) [3, 4, 8, 9].
9. *Zebra retreat *The radiologist retreats from an accurate but unusual diagnosis due to a lack of confidence, despite the presence of supportive evidence. In one respect, this bias is the opposite of the previously described Regret bias (Fig. 19) [3, 6].
10. *Scout neglect *The scout view is the preliminary image taken just before performing the imaging. Ignoring the scout view may result in a diagnostic error (Fig. 20) [4].
11. *Anchoring bias* This type of bias occurs when a radiologist becomes fixated on the first-sight diagnosis, although subsequently presented findings are incompatible with the first diagnosis. Anchoring bias is usually accompanied by confirmation bias, making it more dangerous (Fig. 4) [1, 3–6, 9].
12. *Confirmation Bias* Confirmation bias defines the active searching for more data to confirm the available hypothesis rather than seeking an alternative explanation (Fig. 4) [3, 6, 9].

The types of bias with a summary of the related thought processes of the radiologist are shown in Table 2.

**Table 2:** Radiologists’ thinking processes based on types of bias

Types of bıas	Thınkıng process
Attribution bias	“According to the patient demographics, the diagnosis is X / the diagnosis cannot be Y.”
Alliterative bias (Satisfaction of report)	“Before me, other radiologists have ~ evaluated this study in favor of X. They cannot be wrong.”
Availability Bias	“Just the other day, I saw a patient who had a lesion very similar to this one. If this finding is present, the diagnosis should be X. Their association is always correct.”
Regret bias	“Once, I had overlooked the disease of X. If I miss the possibility of X this time, I may have problems. Let me talk about this in the report.”
Framing Bias	“X is suspected in the clinical note. I should focus on X as the diagnosis to be confirmed or excluded.”
Premature Closure	"My first impression is that it looks like X. So it should be X. It is not necessary to think about other probable diagnoses."
Inattentional bias	"They are investigating X disease. I expect to see the Y finding in this disease." (The obvious Z finding is overlooked)
Hindsight bias	“It is obvious that the patient has X. How could they miss this?” (After the diagnosis is made)
Zebra retreat	"I think I am exaggerating a little. The diagnosis of X is very unusual. Better not to make people laugh at me."
Scout neglect	“There are already cross-sectional images. How can scenogram contribute to the diagnosis? No need to look at it.”
Anchoring Bias	"Against all odds, I think the diagnosis should be X. The existence of the counterarguments does not completely exclude X."
Confirmation Bias	“I believe that the diagnosis is X. I should find previous reports in the literature to support my theory.”

### Possible clinical consequences caused by a radiological error

Errors in the assessment of the imaging examinations of patients are classified by Brady et al. based on the "durations of effect" and "severity of clinical consequences." According to this classification, errors causing no harm are considered "negligible," and errors with minimal ill-effects are accepted as "minor error." "Moderate" and "major errors" are distinguished based on the duration of the negative effects. "Moderate errors" have short-term effects, while "major errors" are defined by long-term undesirable effects. Finally, errors resulting in severe long-term or fatal effects are classified as “extreme errors” [8]. However, it should be kept in mind that this classification is only an example of a method intended to demonstrate how the effects of errors could be evaluated. It is neither a validated nor necessarily an accurate risk assessment tool, and it was not described as an absolute means of classifying the impact of radiological errors.

We can also classify errors according to their effects on patient management. Although some errors have a positive influence on patient management, these are rarely encountered and are the exception (Fig. 3). Apart from those, the most innocent errors are errors that are noticed before causing any effect on patient management. As expected, those errors cause no harm (Fig. 11).

**Fig. 21:**
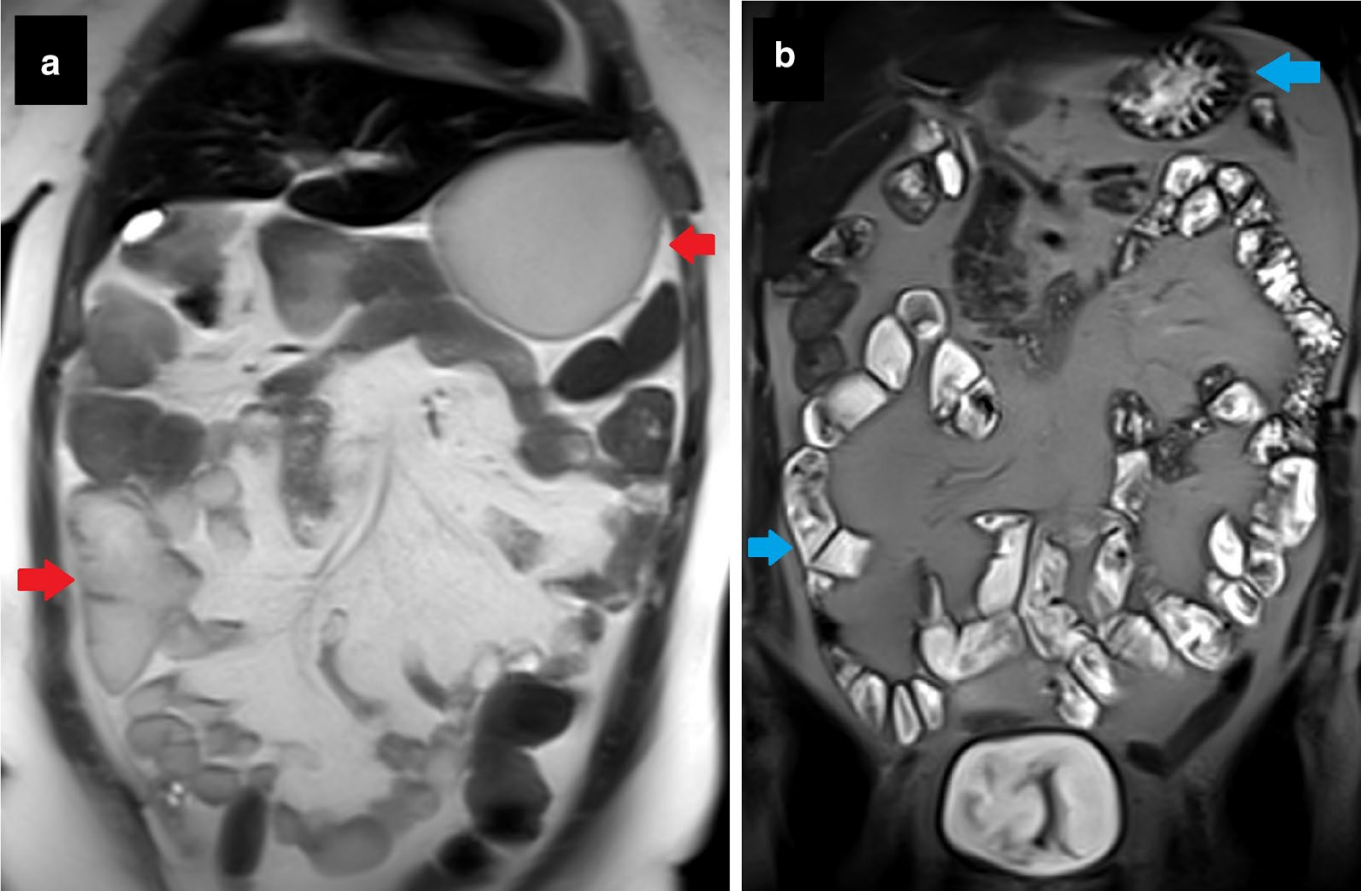
A technique error causing unnecessary effort. A 47-year-old female patient was investigated for inflammatory bowel disease, and MRI enterography was ordered. Before the procedure, the patient had drunk the gadolinium-containing contrast agent in the radiology department by mistake. Due to the paramagnetic feature of gadolinium, the required image quality for evaluation of the intestinal lumen could not be achieved. The MRI technician realized the problem and immediately informed the radiologist. After the radiologist’s evaluation, the study was rescheduled. Although the technical error caused unnecessary effort (repeated examination) in this case, the effective communication between the radiologist and MRI technician enabled them to recognize and correct the error earlier (red arrows, **a** intestinal lumen with gadolinium, blue arrows, **b** intestinal lumen with mannitol in another patient)

**Fig. 22:**
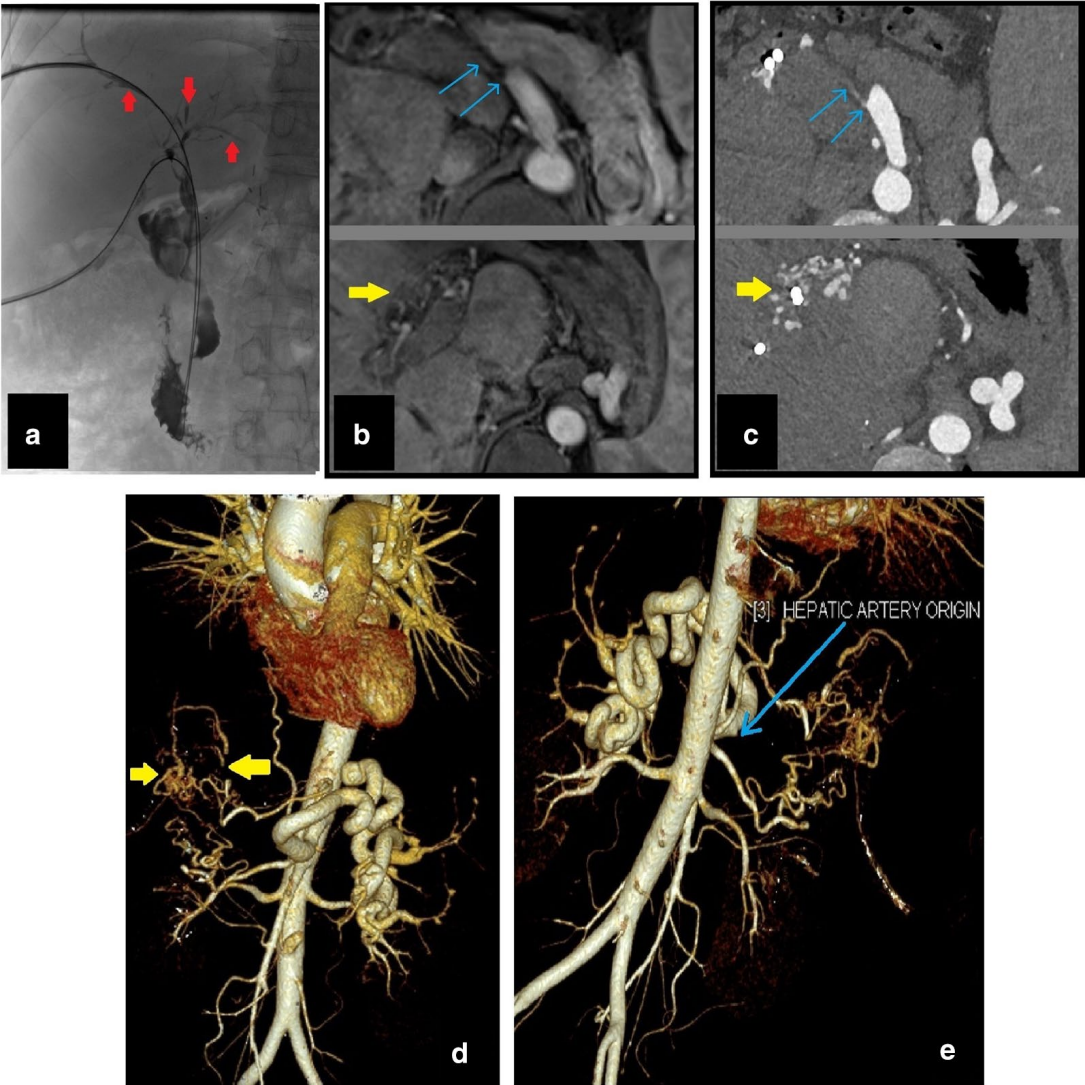
Delay in diagnosis with no apparent harm. A 45-year-old male patient presented at the clinic with jaundice and recurrent cholangitis history after liver transplantation. Due to persistent itching and progressive jaundice, percutaneous transhepatic cholangiography (PTC) was performed for biliary drainage. PTC showed multisegmental involvement with dilated and stenotic biliary ducts (red arrows, **a**). Contrast-enhanced upper abdominal MRI and MRCP for evaluation of the biliary tree were reported as “Chronic cholangiopathic changes due to recurrent episodes of cholangitis” without any differential diagnoses. The clinicians suspected "ischemic cholangiopathy" due to liver transplant history and ordered abdominal CTA to rule out ischemic etiology. The CTA, which was performed two weeks after MRI, revealed hepatic artery occlusion (blue arrows, **c**, **e**) and arterial collateralization (yellow arrows, **c**, **d**). After confirmation of “ischemic biliopathy”, the previous MRI was evaluated retrospectively. The MRI images (**b**) taken from the same slice levels of CTA (**c**) were less obvious to diagnose due to artifacts secondary to inadequate breath-holding. Higher spatial resolution with shorter imaging time of CTA enabled the radiologist to make an accurate diagnosis. As in this case, choosing the wrong technique or modality may decrease the possibility of detecting abnormal findings, cause a delay in diagnosis, and sometimes even make diagnosis impossible. However, in this particular case, the change in the diagnosis did not change the patient management to a great extent and did not cause any apparent harm

Errors that affect patient management can be divided into two groups based on their impact on the patient. In the first group, the error reaches the patient but does not cause any harm. This error type may lead to unnecessary diagnostic effort and further examination (Fig. 21), or it may be clinically insignificant (Fig. 22). In the second group, the error reaches the patient and has a harmful effect. These errors may result in temporary or permanent clinical consequences, either directly or indirectly.

**Fig. 23:**
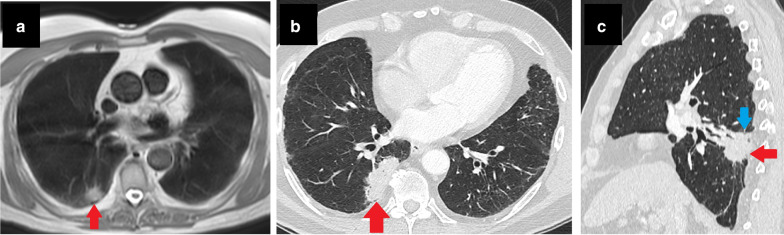
Delay in diagnosis with indirect harm. Cardiac MRI of a 71-year-old male patient for evaluation of angina and left ventricle hypertrophy had shown a hyperintense lesion in the right lung, which had been seen in only one slice of black blood sequence with 8 mm slice thickness (red arrow, **a**). This lesion had been overlooked due to the framing of clinical information and its location. A growing lung mass was detected three years later in the superior segment of the right lower lobe (red arrows, **b**, **c**) on thorax CT. Percutaneous biopsy and subsequent histopathological examination confirmed primary lung cancer. Due to the major fissure invasion developed during the interval period (blue arrow, **c**), the patient underwent right pneumonectomy instead of lobectomy

The consequences of harmful errors can be examined in three groups. The first group is related to misdiagnosis or delay in the diagnosis. Thus, the imaging findings, which are essential for the patient's clinical condition, could be missed or inadvertently interpreted as an inaccurate diagnosis (Fig. 23).

The second group is the errors causing a prolonged hospital stay, which leads to additional follow-up and sometimes the requirement for further treatment. This group of errors either results in acute injury (Fig. 15) or chronic/long-term unwanted situations (Fig. 7).

**Fig. 24:**
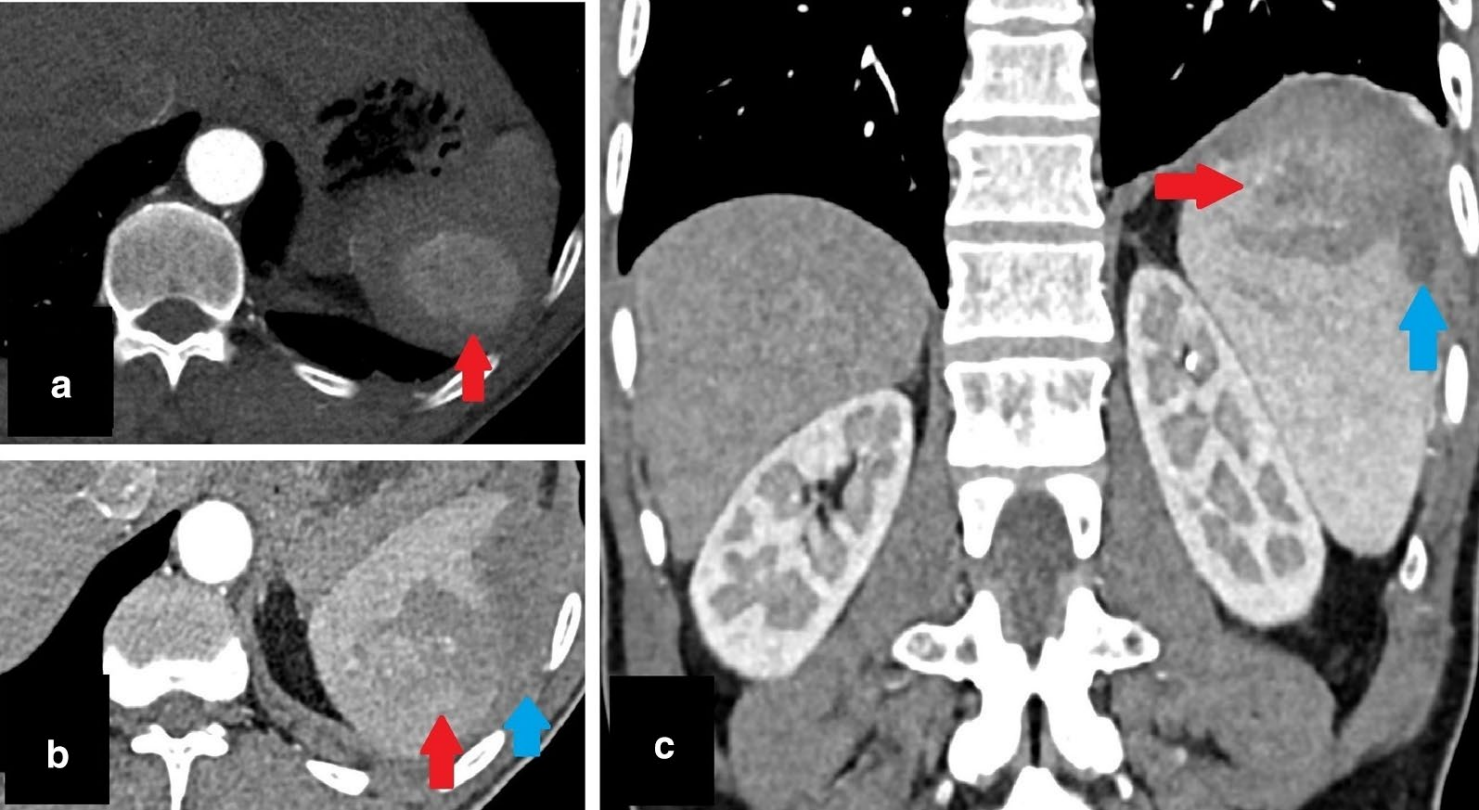
Delay in diagnosis with a life-threatening condition. A 47-year-old male patient presented with complaints of chest pain and abdominal discomfort. History and clinical examination suggested cardiovascular etiology as a possible underlying cause. Coronary CTA was ordered to evaluate the presence of coronary artery disease. The coronary arteries were reported as normal. However, a well-defined splenic hypervascular lesion was missed due to the “edge effect”. The lesion was seen only on the last few slices of “the CT images with a large field of view” (red arrow, **a**). One week later, the patient was admitted to the ER with severe left upper quadrant pain. Abdominal CECT revealed a ruptured lesion (red arrows, B, **c**) and subcapsular splenic hematoma (blue arrows, **b**, **c**) with active extravasation (not shown)

The third group is related to the overlooking or underestimating of significant and life-threatening findings. The latter can result in severe morbidity, even mortality (Fig. 24).

## Conclusion

Errors, discrepancies and confounding biases are integral parts of the daily routine for radiologists and can cause various unexpected clinical consequences. In this study, certain types of radiological errors and biases are explained with case-based examples. By so doing, it is aimed to increase awareness about radiological diagnostic errors and related biases. However, it is obvious that radiological assessment of imaging examinations is a part of overall patient management that may be limited due to the diagnostic utility of the imaging technique and referral information. Therefore, radiological reports should not be expected always to be complete and correct or be regarded as the only tool to catch, confirm, or exclude the diagnosis [1, 3].

**Fig. 25:**
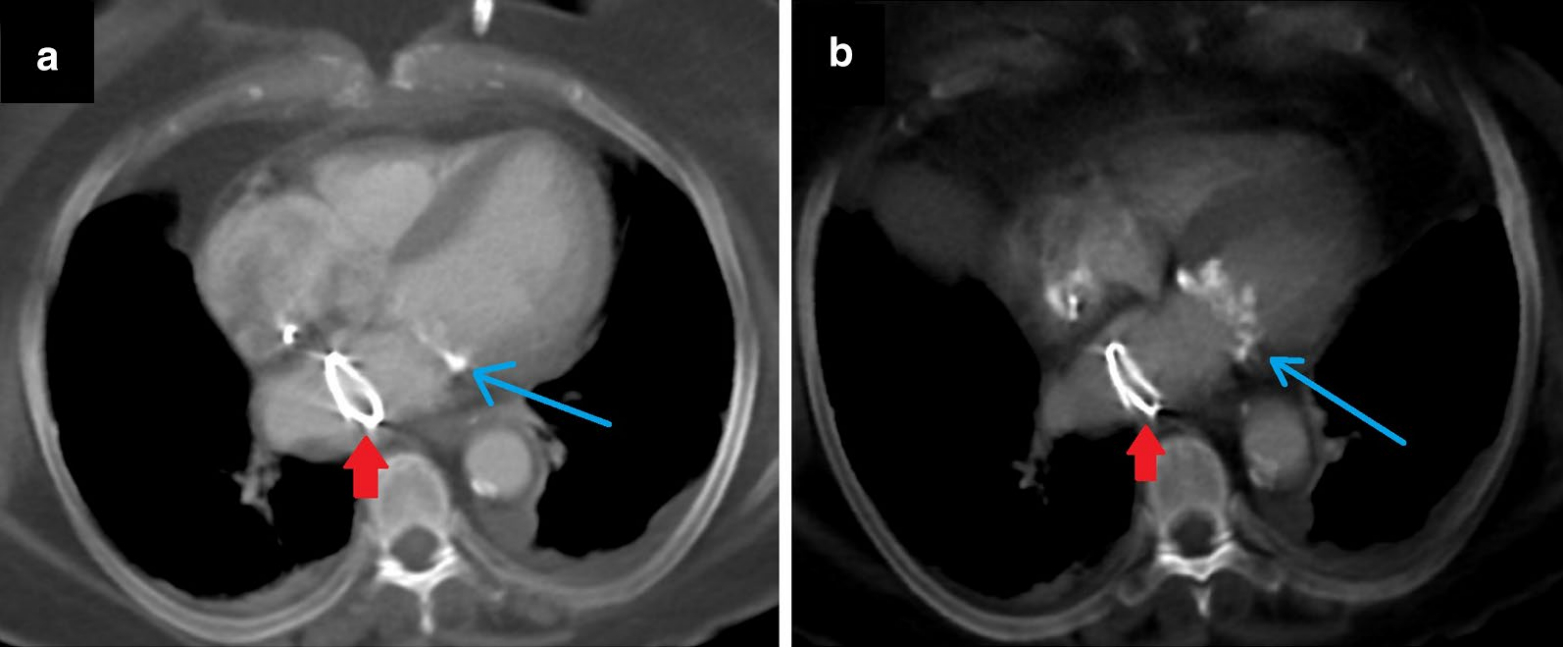
Importance of effective communication. A 65-year-old female patient suffered from the new onset of dyspnea three days after mitral valve replacement. Thorax CT showed the intra-atrial location of the replaced mitral valve (red arrows, **a**, **b**). When the abnormal valve location and new onset of dyspnea were considered, displacement of the replaced mitral valve was suspected. The cardiovascular surgery department was immediately called to report this situation. After a brief opinion exchange with the referrer, it was learned that this valve location is the expected location in patients who have undergone mitral valve replacement with the Chimney technique, which is preferred for patients with extensive mitral annular calcifications (blue arrows, **a**, **b**) to prevent calcified embolism during the procedure. As a result, effective communication between the radiologist and the clinician overcame the possibility of “lack of knowledge and overreading errors”

It should be kept in mind that effective communication between radiologists, radiology technicians, patients and clinicians is one of the key factors in reducing errors and thereby enabling proper patient management (Figs. 11, 21, 25). Finally, awareness of and familiarity with errors and underlying biases is essential for radiologists to be able to cope with them, avoid false interpretations and develop counter-measures [6, 11].

## Data Availability

Data sharing is not applicable to this article as no datasets were generated or analyzed during the current study.

## Abbreviations

CECT: Contrast-enhanced computed tomography
CT: Computed tomography
CTA: Computed tomography angiography
ER: Emergency room
GFR: Glomerular filtration rate
HU: Hounsfield unit
MRCP: Magnetic resonance cholangiopancreatography
MRI: Magnetic resonance imaging
MVA: Motor vehicle accident
NECT: Non-enhanced computed tomography
PTC: Percutaneous transhepatic cholangiography
PTE: Pulmonary thromboembolism
ROI: Region of interest
